# In Situ X-ray Photoelectron Spectroscopic and Electrochemical Studies of the Bromide Anions Dissolved in 1-Ethyl-3-Methyl Imidazolium Tetrafluoroborate

**DOI:** 10.3390/nano9020304

**Published:** 2019-02-22

**Authors:** Jaanus Kruusma, Arvo Tõnisoo, Rainer Pärna, Ergo Nõmmiste, Enn Lust

**Affiliations:** 1 Institute of Chemistry, University of Tartu, Ravila 14A, 50411 Tartu, Estonia; jaanus.kruusma@ut.ee; 2 Institute of Physics, University of Tartu, W. Ostwaldi 1, 50411 Tartu, Estonia; arvo.tonisoo@ut.ee (A.T.); rainer.parna@ut.ee (R.P.); ergo.nommiste@ut.ee (E.N.)

**Keywords:** room temperature ionic liquids, in situ X-ray photoelectron spectroscopy, binding energies, cyclic voltammetry, electrochemical impedance spectroscopy, micro-mesoporous carbon electrode, supercapacitor materials

## Abstract

Influence of electrode potential on the electrochemical behavior of a 1-ethyl-3-methylimidazolium tetrafluoroborate (EMImBF_4_) solution containing 5 wt % 1-ethyl-3-methylimidazolium bromide (EMImBr) has been investigated using electrochemical and synchrotron-initiated high-resolution in situ X-ray photoelectron spectroscopy (XPS) methods. Observation of the Br 3d_5/2_ in situ XPS signal, collected in a 5 wt % EMImBr solution at an EMImBF_4_–vacuum interface, enabled the detection of the start of the electrooxidation process of the Br^−^ anion to Br_3_^−^ anion and thereafter to the Br_2_ at the micro-mesoporous carbon electrode, polarized continuously at the high fixed positive potentials. A new photoelectron peak, corresponding to B–O bond formation in the B 1s in situ XPS spectra at *E* ≤ −1.17 V, parallel to the start of the electroreduction of the residual water at the micro-mesoporous carbon electrode, was observed and is discussed. The electroreduction of the residual water caused a reduction in the absolute value of binding energy vs. potential plot slope twice to ca. d*BE* d*E*^−1^ = −0.5 eV V^−1^ at *E* ≤ −1.17 V for C 1s, N 1s, B 1s, F 1s, and Br 3d_5/2_ photoelectrons.

## 1. Introduction

Electricity is one of the most convenient modes of energy that can be very easily converted into other forms of energy. Besides the flexibility, the electrical energy does not pollute the surrounding environment and the electrical devices are small and quiet. Therefore, electricity has found applications in many fields of modern technology including electrochemical power sources, electrosynthesis, and galvanic processes. In mobile applications and isolated places, electrical devices should have high specific energy and power density. In terms of environmental protection and sustainability, i.e., the recycle economy point of view, electrical energy generating devices should be reusable, i.e., rechargeable. 

Two types of reusable powerful electrical energy storage systems are known and applied: rechargeable electrochemical faradic cells and supercapacitors [1,2,3,4,5,6,7,8,9,10,11]. Supercapacitors are characterized by very high specific power density and capacitance values (up to 175 F g^−1^ for aqueous and up to 100 F g^−1^ for nonaqueous electrolyte-based, commercial electrochemical double layer capacitor (EDLC) cells [1,2,3], and from 120 to 150 F g^−1^ for novel micro-mesoporous carbon electrode-based systems in nonaqueous electrolytes [11,12,13,14,15,16,17,18]). The number of recharging cycles that can be applied exceeds 500,000, more than 100 times higher than the corresponding number of rechargeable electrochemical cells [1,19,20]. However, the energy density of supercapacitors is much lower than that for faradic electrochemical cells [1,6]. Therefore, it seems to be very attractive to combine the superior properties of both types of electrical energy storage systems into a common device, a so-called hybrid capacitor, where the electrical double layer charging is combined with the fast reversible redox process(es) taking place at the faradic-type electrode [1,3,4]. Different redox couples (e.g. hydroxyquinone/quinone, Ru/RuO_2_, MnO(OH)/MnO_2_, I^−^/I_2_, metal chalcogenides, etc.) have been studied for the construction of hybrid capacitors [1,3,4]. Various electrolytes, based on aqueous or non-aqueous solutions, liquid or solid, have been tested as well [1,3,4,19,20,21,22,23,24,25,26,27,28,29]. It should be noted that the applicable cell potential (Δ*E*) is moderate and limited (ca. 1.2…1.6 V) for the aqueous electrolyte-based hybrid capacitors due to electrochemical decomposition of water at Δ*E* ≥ 1.2 V [3,20,21]. Therefore, non-aqueous electrolytes, having a wider applicable Δ*E* range, are more desirable for the construction of hybrid supercapacitors due to the higher energy density stored [1,2,3].

The properties of iodide anions containing non-aqueous electrolyte systems have been studied in various electrodes [30,31,32,33,34,35,36]. Remarkably, high series capacitance values (more than 120 mF cm^−2^) have been measured for pyrolytic graphite — 5 wt % 1-ethyl-3-methylimidazolium iodide (EMImI) dissolved in a 1-ethyl-3-methylimidazolium tetrafluoroborate (EMImBF_4_) interface (*E* > 0.5 V vs. Ag/AgCl) [33]. Very high specific capacitance values (up to 245 F g^−1^ at the Δ*E* = 1.0 V) have been measured for a D-glucose-derived, micro-mesoporous carbon-based EDLC [35]. However, for a 5 wt % EMImI solution in EMImBF_4_, the nearly reversible I^−^ anion adsorption takes place only at Δ*E* ≤ 2.4 V. Within the cell potential range from 2.6 to 3.0 V, the complicated mixed kinetic faradic processes dominate, decreasing the EDLC reversibility and the energetic efficiency of the device [35].

Yamazaki et al. [37] investigated the capacitive properties of 1.0 M 1-ethyl-3-methylimidazolium bromide (EMImBr) dissolved in EMImBF_4_ at the activated carbon fiber cloth electrode. Charging this system up to Δ*E* = 2.0 V (at the gravimetric current density (*i_g_*) *i_g_* = 100 mA g^−1^), a 59.0 F g^−1^ specific capacitance was obtained. It is obvious that this specific capacitance value is much lower than that for the I^−^ anion-based system, as the specific adsorption of Br^−^ anion is much weaker than that of the I^−^ anion [35] However, the bromide anion-containing system possessed excellent cycleability and a coulombic efficiency up to Δ*E* = 2.0 V [37]. 

Adsorption of the Br^−^ anion dissolved in EMImBF_4_ at the Bi(111) electrode has demonstrated better reversibility compared to I^−^ anion adsorption [38]. These data indicate that the hybrid EDLCs filled with non-aqueous electrolytes containing the Br^−^ anion could have higher energy efficiencies than devices based on the I^−^ anion.

Quite recently, Gastrol et al. [39] published a work where the capacitance of the bromide anion containing a hybrid EDLC was extended up to 314 F g^−1^ at Δ*E* = 1.1 V by the partial oxidation of Br^−^ anion (in 2 M KOH aq. solution) to the BrO_3_^−^ anion. However, the Δ*E* for the studied system was limited by the start of the electrochemical decomposition of water at the positively charged activated carbon electrode [39].

Therefore, due to the limited amount of information characterizing the electrochemical properties of the bromide anion containing room temperature ionic liquid electrolytes at micro-mesoporous carbon electrodes, the micro-mesoporous molybdenum carbide-derived carbon (C(Mo_2_C) electrode in a 5 wt % EMImBr solution in EMImBF_4_ was studied using cyclic voltammetry (CV), electrochemical impedance spectroscopy (EIS), and synchrotron radiation-initiated high-resolution in situ X-ray photoelectron spectroscopy (XPS) methods. 

## 2. Materials and Methods 

The in situ XPS spectra were recorded at the polarized Mo_2_C derived carbon (C(Mo_2_C)) electrodes under high vacuum at the synchrotron-initiated adjustable energy X-ray beamline I411, Max II Laboratory, Lund University (Lund, Sweden). 

C(Mo_2_C) electrodes (the working electrode, WE), covered with a very thin layer of a 5 wt % EMImBr (≥99%, Iolitec Ionic Liquids Technologies, Heilbronn, Germany) solution in EMImBF_4_ (≥99.0% (HPLC), Fluka, Honeywell, Bucharest, Romania), containing less than 200 ppm water, were studied. The lower part of the WE was soaked and kept during the XPS measurement in room temperature ionic liquids (RTILs) containing a reservoir. The 5 wt % EMImBr solution in EMImBF_4_ was prepared and stored in an Ar-filled glove box containing less than 0.1 ppm water and oxygen. The C(Mo_2_C) electrode was positioned almost vertically, polarized during the in situ XPS experiments using a three-electrode electrochemical cell. Platinum gauze (with an apparent area of ca. 2 cm^2^ and a grid size of 100 mesh, 99.9%, Merck KGaA, Darmstadt, Germany) was used as the counter electrode (CE), and Ag wire covered with AgCl (Ag/AgCl in pure EMImBF_4_ RTIL) was used as the reference electrode (RE) in RTIL.

**Figure 1:**
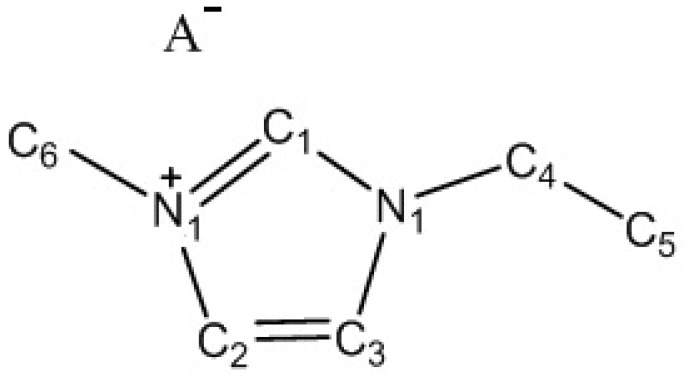
Notation of the carbon and nitrogen atoms of 1-ethyl-3-methylimidazolium (EMIm^+^) cation. A^−^ marks the Br^−^ or BF_4_^−^ anion, respectively.

The design of the synchrotron radiation beamline, the working, counter, and reference electrodes, and the used electrochemical cell have been described in detail [40]. The reproducibility data for the synchrotron radiation-initiated in situ XPS measurements for C 1s, N 1s, F 1s, and B 1s signals have been published as well [40]. The stability of the binding energy (*BE*) peak value for the C 1s signal was ±0.12 eV. The number of independent experiments was 17, and the relative standard deviation was only ±0.041% over 9.5 h of test time [41]. The measured amounts of detected photoelectrons, forming XPS peaks, had the following stabilities: for aliphatic carbon (C_5_, Figure 1) C 1s, RSD = ±8.8 %, for B 1s, RSD = ± 3.4 %, for N 1s, RSD = ± 6.2 %, and for F 1s, RSD = ±7.6 % [40]. For additional supporting electrochemical measurements (inside the very dry Ar-filled glove-box), a carbon fiber (*d* = 11 μm, Bioanalytical Systems, Inc., West Lafayette, IN, USA) microelectrode was used.

For a better understanding and a more detailed discussion of the collected in situ XPS data, the elements, forming the EMIm^+^ cation, were numbered, as shown in Figure 1. XPS data analysis and *BE* spectrum fitting procedures were performed using IgorPro (ver. 6.2.2.2, WaveMetrics, Inc., Lake Oswego, OR, USA) and CasaXPS (ver. 2.315, Casa Software Ltd., Teignmouth, UK) software, respectively [40]. The C 1s XPS spectra were fitted using the four photoelectron (PE) peak model, where one C 1s PE peak was related to the aliphatic carbon (C_5_, Figure 1), and the other three C 1s PE peaks were related to the hetero-aromatic ring (included or bounded) carbons (C_1_, C_2_, C_3_, C_4_, and C_6_, as noted in Figure 1) in the same manner as it was described earlier by Foelske-Schmitz et al. [42] and Licence et al. [43,44] using a combined Gaussian–Lorentz function with ratio 70:30, respectively. The full width at half maximum (FWHM) of the C 1s PE peak, related to a so-called hetero-aromatic carbon, was allowed to change between 0.9 and 1.1 eV, as described by Licence et al. [43]. The FWHM for the C 1s PE peak related to aliphatic carbon (C_5_) was unfixed. The other three PE peak positions were fixed relative to each other. The *BE* of C_2_ was equal to the *BE* of C_3_, and the *BE* of C_4_ was equal to the *BE* of C_6_. *BE* separation between C_2_ and C_1_ was fixed to 0.70 eV, and *BE* separation between C_2_ and C_4_ was fixed to 0.50 eV. The *BE* of C_5_, representing the carbon atoms in the alkyl chain (having sp^3^ electronic configuration), was fixed to 285.3 eV based on the non-polarized C(Mo_2_C) WE data. The PE peak areas for C_1_, C_2_+C_3_, and C_4_+C_6_ carbons were fixed at a ratio of 0.5:1:1 [42].

The N 1s, B 1s, F 1s, and Br 3d X-ray photoelectron spectra were fitted using the same ratio of the Gaussian–Lorentz function as described for C 1s XPS, leaving the FWHM and peak positions free. Later, the obtained individual peak *BE* was corrected according to the *BE* of the C_5_ carbon measured at fixed potential.

The ratios of the XPS PE peaks have been calculated using the following equation:

$$X_{x,}\%=100\times \frac{A_{x}}{\sum_{i=1}^{n}A_{i}}$$

where *X_x_* is the ratio of the PE peak “*x,*” and *A_x_* is the amount of the counted PE (i.e., the PE peak area) of the XPS signal “*x,*” corrected with the synchrotron ring current value and the number of scans.

In addition to XPS studies, cyclic voltammetry (CV) and electrochemical impedance spectroscopy (EIS) measurements were performed in the oxygen and humidity-free Ar-filled glovebox. A Gamry Instruments “Reference 3000” potentiostat, controlled by the Gamry Instruments “Framework” (ver. 6.32) software, was used to polarize the electrodes. The potential sweep rate of 1.0 mV s^−1^ and the potential step of 5 mV were used for obtaining the CVs. The EIS measurements were performed in potentiostatic mode (vs. the RE potential), and the ac modulation amplitude was 5.0 mV. A single sinusoidal potential wave was used for modulation of the C(Mo_2_C) electrode potential. The XPS and electrochemical measurements were conducted at 22 °C. 

## 3. Results and Discussion

### 3.1. Characteristic Changes in C 1s, N 1s, B 1s, F 1s, and Br 3d In Situ X-ray Spectra Obtained at the Negatively Polarized C(Mo_2_C) Electrode 

**Figure 2:**
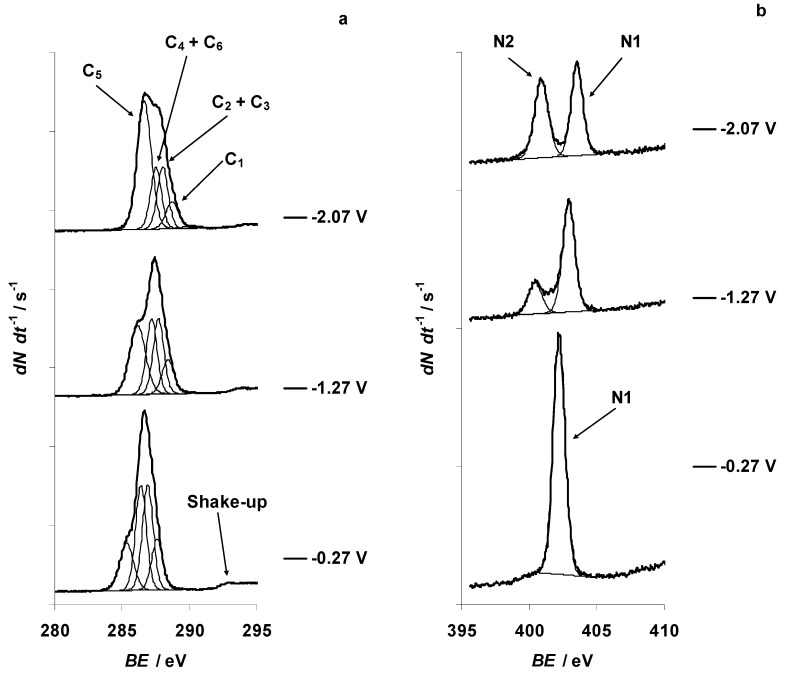

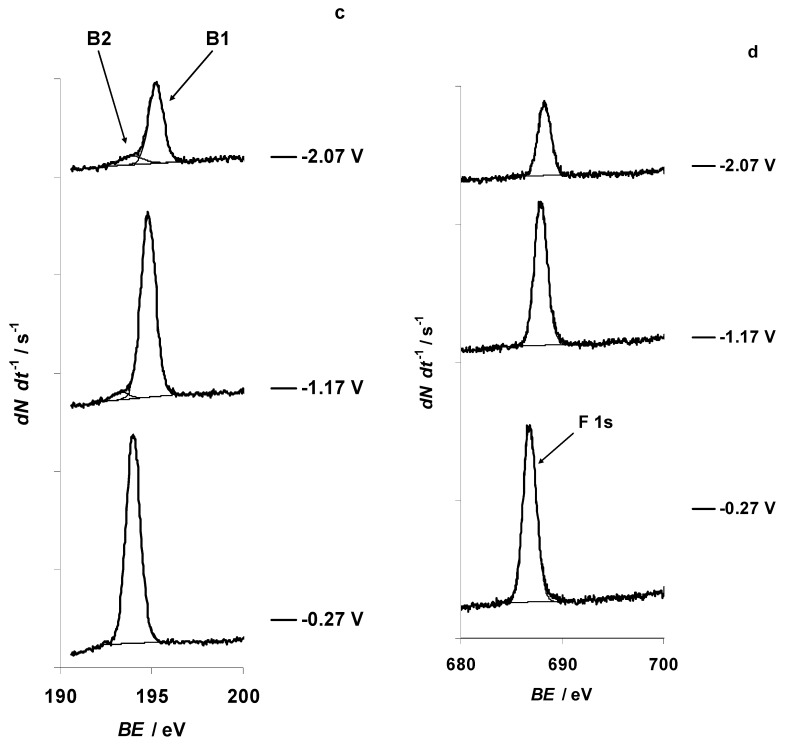

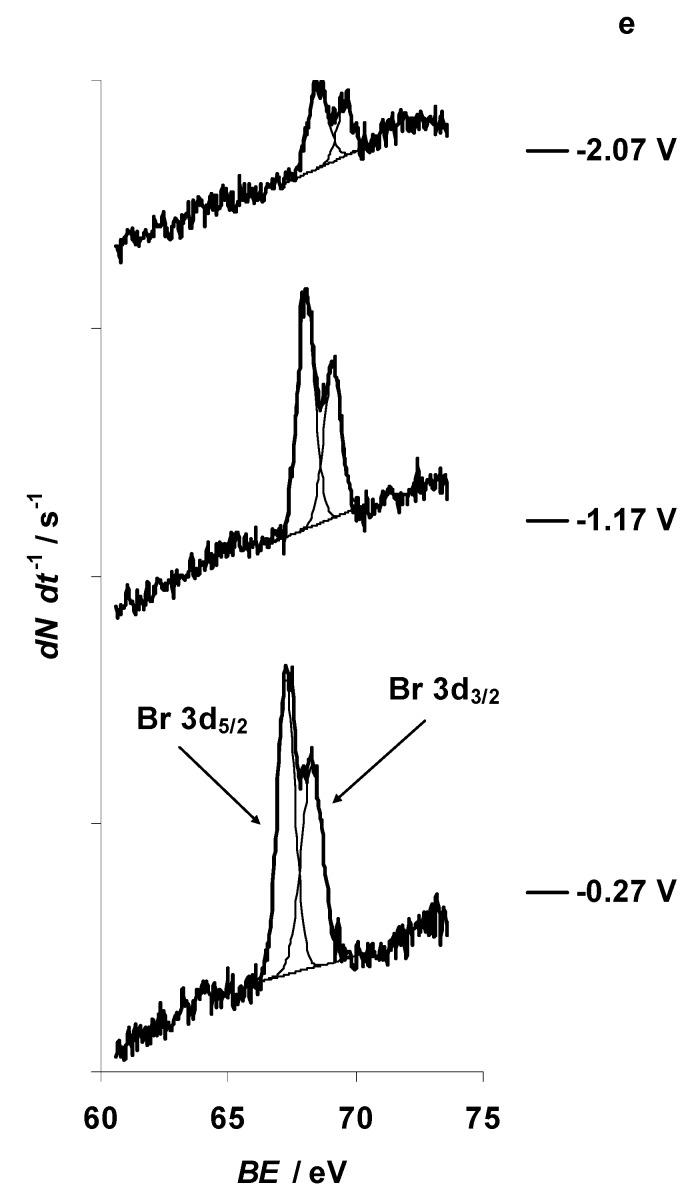
C 1s, N 1s, B 1s, F 1s, and Br 3d X-ray photoelectron (PE) spectra for the 5 wt % EMImBr solution in EMImBF_4_ measured at the fixed negative potentials of the C(Mo_2_C) electrode. The binding energy (*BE*) scales for the XPS experiments have been referenced to the *BE* of the C 1s photoemission line related to aliphatic carbon (*BE* = 285.3 eV) measured for the non-polarized and grounded electrode. (**a**) C 1s PE spectra, fitted applying four C 1s photoelectron peak model (regular lines) at various potentials noted in the figure; excitation energy was 400 eV, and the PE signal intensity (d*N* d*t*^−1^) scale between tick marks was 100 counts s^−1^. (**b**) N 1s PE spectra; excitation energy was 500 eV, and the PE signal intensity scale between tick marks was 35 counts s^−1^. (**c**) B 1s PE spectra; excitation energy was 250 eV, and the PE signal intensity scale between tick marks was 20 counts s^−1^. (**d**) F 1s PE spectra; excitation energy was 800 eV, and the PE signal intensity scale between tick marks was 8 counts s^−1^. (**e**) Br 3d PE spectra; excitation energy was 120 eV, and the PE signal intensity scale between tick marks was 20 counts s^−1^.

**Figure 3:**
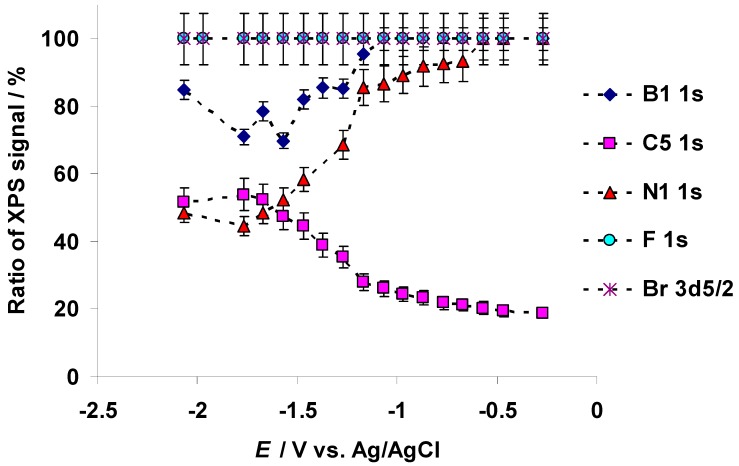
Dependence of EMIm^+^ “aliphatic” carbon C 1s (marked in the figure as C5), imidazolium nitrogen atoms N 1s (N1), BF_4_^−^ anion boron B 1s (B1), BF_4_^−^ anion fluorine F 1s, and Br^−^ anion bromine Br 3d_5/2_ photoelectron peaks ratios for the 5 wt % EMImBr solution in EMImBF_4_ at various C(Mo_2_C) electrode negative potentials.

The X-ray photoelectron spectra for C 1s, N 1s, B 1s, F 1s, and Br 3d PEs at fixed negative potentials were recorded within the potential range from −2.07 to −0.27 V and are presented in the Figure 2a–e, respectively. Exact positions of the PE peaks are presented in Table S1. The PE spectra indicate significant changes for the aliphatic carbon (C_5_) C 1s (Figure 2a and Figure 3) and nitrogen (N1) N 1s (Figure 2b and Figure 3) XPS signals at *E* = −1.27 V. For N 1s XPS, a very small new PE peak formed at *E* = −0.67 V (located at *BE* = 400.3 eV and marked hereafter as N2, not shown for space constraints), causing a very small deviation in the relative amount of N1 1s signal (Figure 3). A shift of the C(Mo_2_C) electrode potential toward more negative values caused a noticeable increase in the relative size of the N2 1s PE peak. At *E* = −1.17 V, a small shoulder formed in the initial B 1s PE peak (at *BE* = 194.8 eV, defined as B1 hereafter and in Figure 2c and Figure 3). Parallel to this, gas bubbles started to form occasionally at the C(Mo_2_C) working electrode surface. This shoulder corresponded to a new B 1s PE peak (defined as B2 hereafter and in Figure 2c) with *BE* = 193.3 eV. The initial B 1s XPS peak (B1) was located at *BE* = 194.8 eV (*E* = −1.17 V), based on the NIST XPS database [45]. The new B 1s PE peak (B2) could correspond to the formation of some sort of boron–oxygen compound. 

No changes in the shapes of the F 1s (Figure 2d and Figure 3) and Br 3d (Figure 2e and Figure 3) XPS signals were noted, indicating high electrochemical stability of these elements within the negative electrode potential range. The decrease in intensities of the B 1s, F 1s, and Br 3d XPS signals, notable in Figure 2c–e, could be explained by the decrease in the concentration of Br^−^ and BF_4_^−^ anions at the ionic liquid–carbon surface due to electrostatic repulsion of anions from the negatively charged C(Mo_2_C) electrode.

Analysis of the relationship between the C_5_ 1s, N1 1s, B1 1s, F 1s, and Br 3d_5/2_ PE BEs upon the potential applied to micro-mesoporous C(Mo_2_C) electrode indicated that d*BE* d*E*^−1^ = −1 eV V^−1^ for C_5_ 1s, N1 1s, B1 1s, F 1s, and Br 3d_5/2_ within the potential range from −1.17 to 1.23 V (the d*BE* d*E*^−1^ slope values are presented in Table S2, given in the supplementary information). However, for the potential range from −2.07 to −1.17 V, d*BE* d*E*^−1^ = −0.5 eV V^−1^ for C_5_ 1s, N1 1s, B1 1s, F 1s, and Br 3d_5/2_ PEs. Therefore, the twofold decrease in the d*BE* d*E*^−1^ slope, the formation of the gas bubbles at the C(Mo_2_C) electrode and the new shoulder into the initial B 1s PE peak, and the formation of a B–O bond at *E* = −1.17 V were initiated by reduction processes at the electrode surface.

### 3.2. The Electrochemical Measurements Data at Negatively Polarized C(Mo_2_C) Electrode

**Figure 4:**
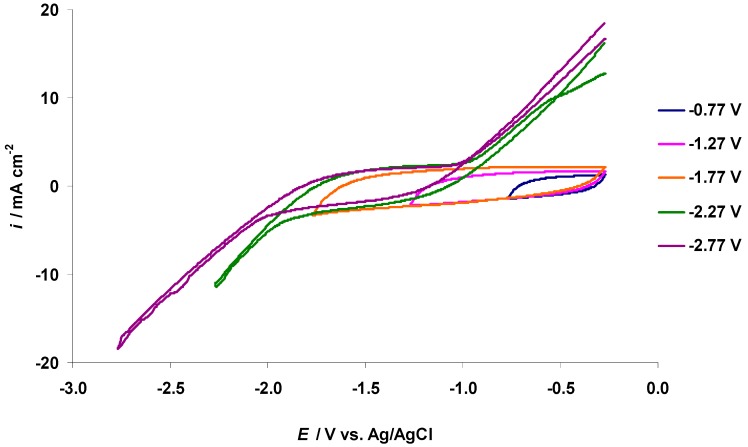
Cyclic voltammetry (CV) curves for negatively polarized C(Mo_2_C) electrode in the 5 wt % EMImBr solution in EMImBF_4_ measured at variable potential ranges and normal pressure in the Ar-filled glove box conditions (second CV scans are presented starting and ending at *E* = −0.27 V, and the potential scan rate was 1.0 mV s^−1^ ).

**Figure 5:**
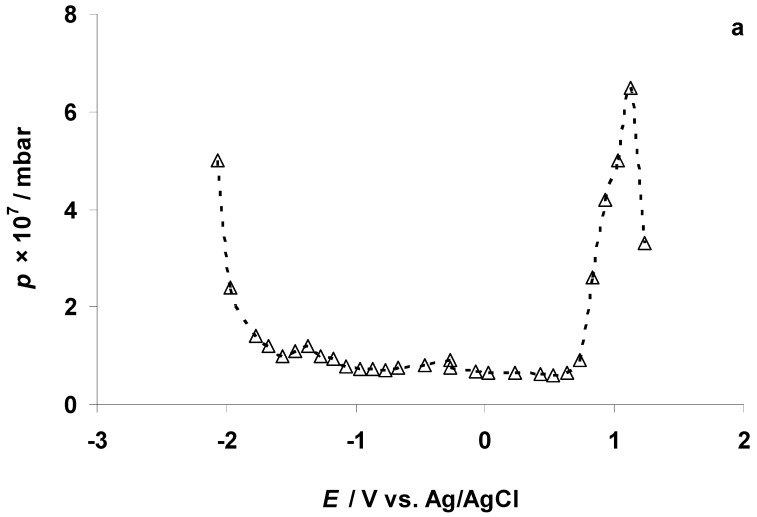

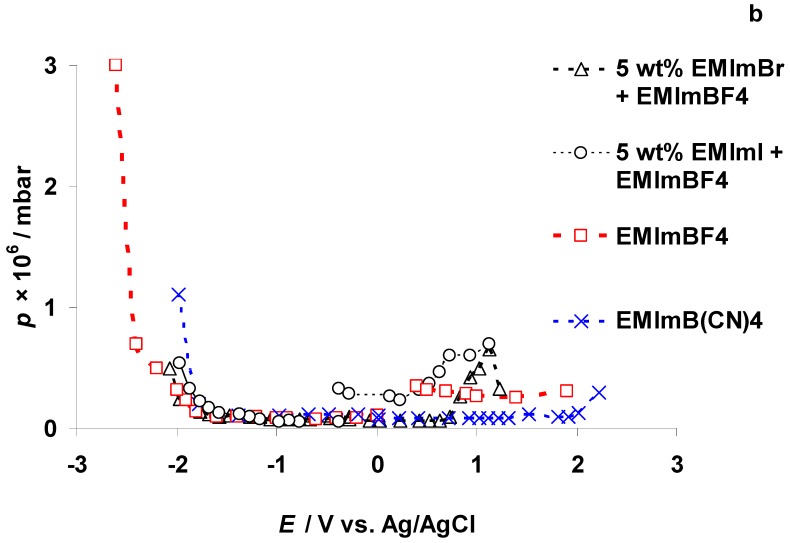
The dependence of gas pressure, *p* (measured inside the vacuum chamber of the X-ray photoelectron spectrometer), on the negative and positive potentials applied to the C(Mo_2_C) electrode soaked in (**a**) the 5 wt % EMImBr solution in EMImBF_4_ and (**b**) the 5 wt % EMImBr solution in EMImBF_4_ (△), EMImB(CN)_4_ (×), EMImBF_4_ (□), and the 5 wt % EMImI solution in EMImBF_4_ (○).

The cyclic voltammetry (CV) curves for the negatively polarized micro-mesoporous C(Mo_2_C) electrode soaked in the 5 wt % EMImBr solution in EMImBF_4_ at various fixed potential sweep regions are shown in Figure 4. Intensive electroreduction of the imidazolium cation [13,40,46,47] started at *E* = −1.90 V, parallel to the remarkable increase in the pressure in the XPS measurement chamber (Figure 5a). (The behavior of the XPS chamber pressure at the positive C(Mo_2_C) electrode potentials is explained later in the text.) In Figure 5b, the XPS vacuum chamber pressure values, containing also our previously published data [40,46,47], show that intensive electroreduction processes started at comparable negative potentials, depending only weakly on the chemical composition of the anions in the electrolyte solution.

**Figure 6:**
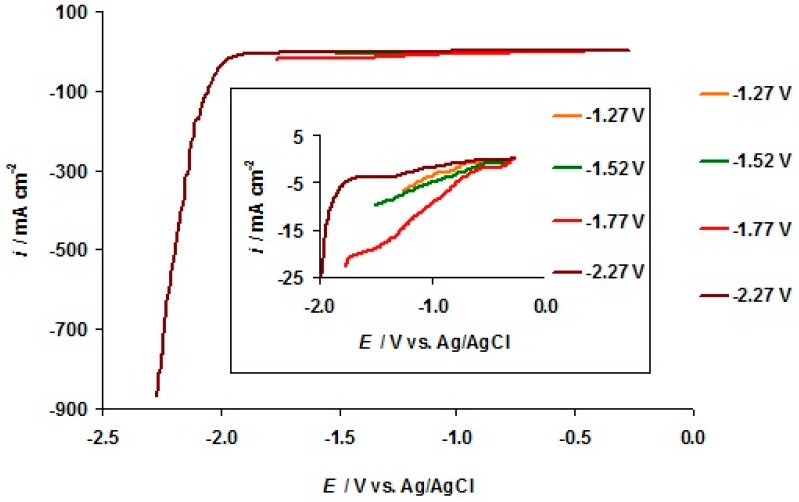
Potential linear sweep (LS) data recorded for negatively polarized C fiber microelectrode soaked in the 5 wt % EMImBr solution in EMImBF_4_. Second sweeps moving toward more negative potentials are shown, measured at the normal pressure in the Ar-filled glove box conditions. Potential sweeps started at *E* = −0.27 V and ended at the potentials indicated in the figure; the potential scan rate was 1.0 mV s^−1^. The inset represents the zoomed in part of the LSs shown above.

For more detailed analysis of the electrochemical processes in the 5 wt % EMImBr solution in EMImBF_4_, potential linear sweep measurements were performed at the carbon fiber microelectrode (d = 11 ± 2 μm). The carbon fiber microelectrode was selected to suppress the “masking” effect of the electrical double layer charging capacitive current during the potential sweep and to have more effective mass transport of possible reagents to the electrode surface. Data of the second, more negative potential values moving toward sweep shows that the intensive electrochemical reduction process started in the 5 wt % EMImBr solution in the EMImBF_4_–C interface only at *E* < −1.90 V, where nearly an exponential increase in current density takes place (Figure 6, brown line). The amplified low current density section in Figure 6, brown line, indicates that a slight increase in current density starts at *E* = −1.74 V followed by the most intensive increase at *E* = −1.90 V. This slight increase in the current might indicate the adsorption of the EMIm^+^ cations before the start of the electroreduction of EMIm^+^ cations. It is also interesting to note that the reduction current densities at *E* > −1.97 V are much lower for the potential sweep curve collected from −0.27 to −2.27 V than the currents for the potential sweeps performed before, and stopped at *E* ≥ −1.77 V. This phenomenon could be explained by partial passivation of the carbon fiber microelectrode within the potential range −2.27 V < *E* < −1.77 V during the first potential sweep (not shown for clearance) due to the irreversible reduction of EMIm^+^ cations and the formation of the dielectric EMIm–EMIm dimer film at the carbon electrode [13]. However, for the potential sweeps conducted within the potential ranges from −0.27 to −1.27 V (orange line), from −0.27 to −1.52 V (green line), and from −0.27 to −1.77 V (violet line), there was a gradual electrochemical activation of the 5 wt % EMImBr solution in the EMImBF_4_–C microelectrode interface (Figure 6). 

**Figure 7:**
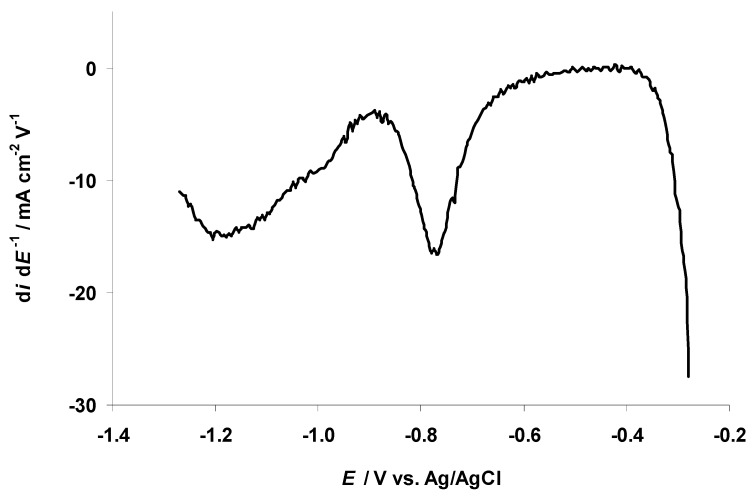
Differentiated potential linear sweep curve (d*i* d*E*^−1^) recorded for negatively polarized C fiber microelectrode soaked in the 5 wt % EMImBr solution in EMImBF_4_. The second sweep was shown measured at the normal pressure in the Ar-filled glove box conditions starting at *E* = −0.27 V and ending at *E* = −1.27 V, with a potential scan rate of 1.0 mV s^−1^.

The d*i* d*E*^−1^ curve, constructed using the potential linear sweep data collected within the potential range from −0.27 to −1.27 V, shows a d*i* d*E*^−1^ peak (d*i* d*E*^−1^ = −16.5 mA cm^−2^ V^−1^) at *E* = −0.78 V (Figure 7), marking the occurrence of an electroreduction process. The curve passes through a minimum at *E* = −0.89 V. A new, wide negative current maximum has a peak value (−15.0 mA cm^−2^ V^−1^) at *E* = −1.19 V (Figure 7). This second, very wide negative current maximum is likely caused by the very slow electroreduction of the residual water at the carbon electrode. The electrochemical reduction of the residual water at the glassy carbon electrode was reported by Cheek et al. [48].

The reduction of the d*BE* d*E*^−1^ slope to −0.5 eV V^−1^ at *E* ≤ −1.17 V for C 1s, N 1s, B 1s, F 1s, and Br 3d_5/2_ PE signals (Table S2), the start of the low intensity formation of gas bubbles (hydrogen formation and evolution) at the C(Mo_2_C) electrode, and the formation of a new shoulder into the B 1s PE peak (Figure 2c and Figure 3) indicated the formation of a B–O bond at *E* = −1.17 V, initiated by the electrochemical reduction of the residual water (210 ppm, based on the Karl Fisher titration method) in the 5 wt % EMImBr solution in EMImBF_4_. We suppose that the reduction of d*BE* d*E*^−1^ slope to the value ca. −0.5 eV V^−1^ was caused by the chemisorption [49,50] of partially hydrolyzed EMImBF_4_, which is more hydrophilic due to the B–O bonds formed at the C(Mo_2_C) electrode surface at *E* ≤ −1.17 V (Table S2).

The CV data for the 5 wt % EMImBr solution in the EMImBF_4_–C microelectrode interface (Figure 6 and Figure 7) are in an agreement with the changes noted for C_5_ 1s, N 1s, and B 1s XPS data (Figure 2a–c and Figure 3). The formation of a boron–oxygen bond at *E* = −1.17 V and the stability of the B2 1s PE peak at more negative C(Mo_2_C) potentials confirm the electrochemical reduction of the residual water from the 5 wt % EMImBr solution in EMImBF_4_. 

However, the formation of the B–O bond only at *E* ≤ −1.17 V indicated the chemical and electrochemical stability of the BF_4_^−^ anion at less negative potentials in the presence of residual water. Thus, the formation of a new B 1s PE peak at ca. *E* = −1.1 V could be used as an indicator of the presence of the residual water in the electrochemical system.

**Figure 8:**
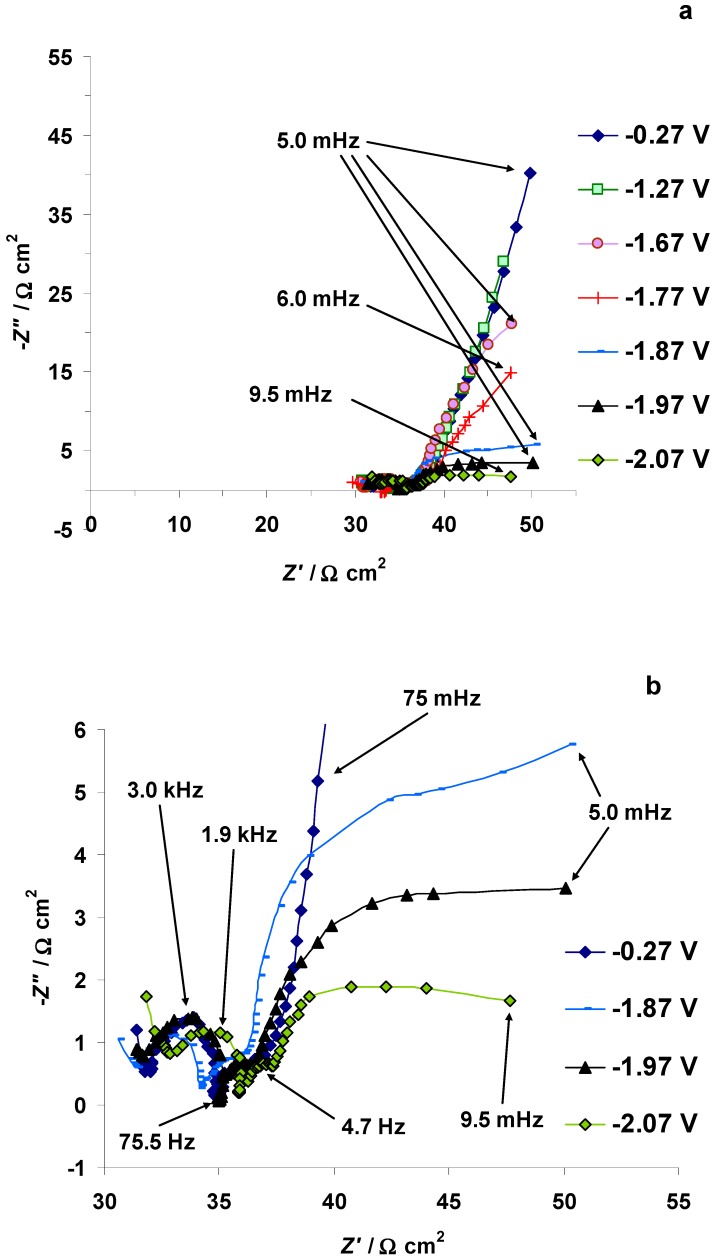
Electrochemical impedance spectroscopy Nyquist plots measured for the 5 wt % EMImBr solution in the EMImBF_4_–C(Mo_2_C) system (**a**) at variable C(Mo_2_C) electrode negative potentials and (**b**) at selected C(Mo_2_C) electrode negative potentials where intensive EMIm–EMIm dimer formation has started. *Z*′ and *Z*″ mark the real and imaginary parts of the electrochemical impedance, respectively.

The electrochemical impedance spectroscopy (EIS) data, i.e., Nyquist plots measured in the potentiostatic regime for the 5 wt % EMImBr solution in the EMImBF_4_–C(Mo_2_C) interface, have a stable characteristic shape from *E* = −0.27 to *E* = −1.67 V. Nyquist plots consist of a high-frequency semicircle (caused by the restricted mass transport in the micro-mesoporous C(Mo_2_C) electrode) followed by a semi-vertical line formed at medium and low frequencies, demonstrating the slow adsorption of the RTIL ions at the energetically inhomogeneous micro-mesoporous C(Mo_2_C) electrode surface. At *E* = −1.77 V, a small inductive loop forms at the end of the small high frequency semicircle, and the slope of the low frequency line starts to reduce (Figure 8a,b). The Nyquist plots indicate also that, at *E* ≤ −1.87 V, the imaginary values of the impedance (*Z*″) become less negative at low frequencies, and the low-frequency part of the Nyquist plot initially becomes almost parallel with the axis of the real part of the impedance (*Z*′), indicating the slow charge transfer step-limited process. 

At *E* ≤ −2.07 V, a new low-frequency arc forms, indicating the existence of the mixed kinetic charge transfer and adsorption step-limited processes (Figure 8b). Parallel to the formation of the arcs in the low-frequency range, the Nyquist plots became noisier, indicating the formation of gaseous substances and/or an unstable dielectric film at the C(Mo_2_C) electrode surface [40,46,47]. 

**Figure 9:**
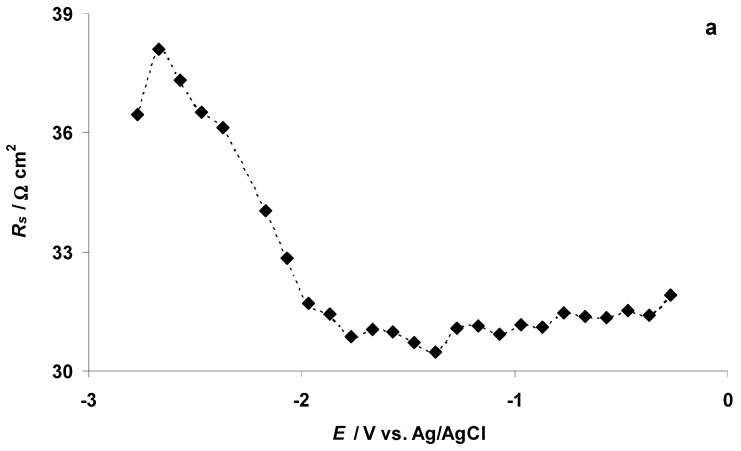

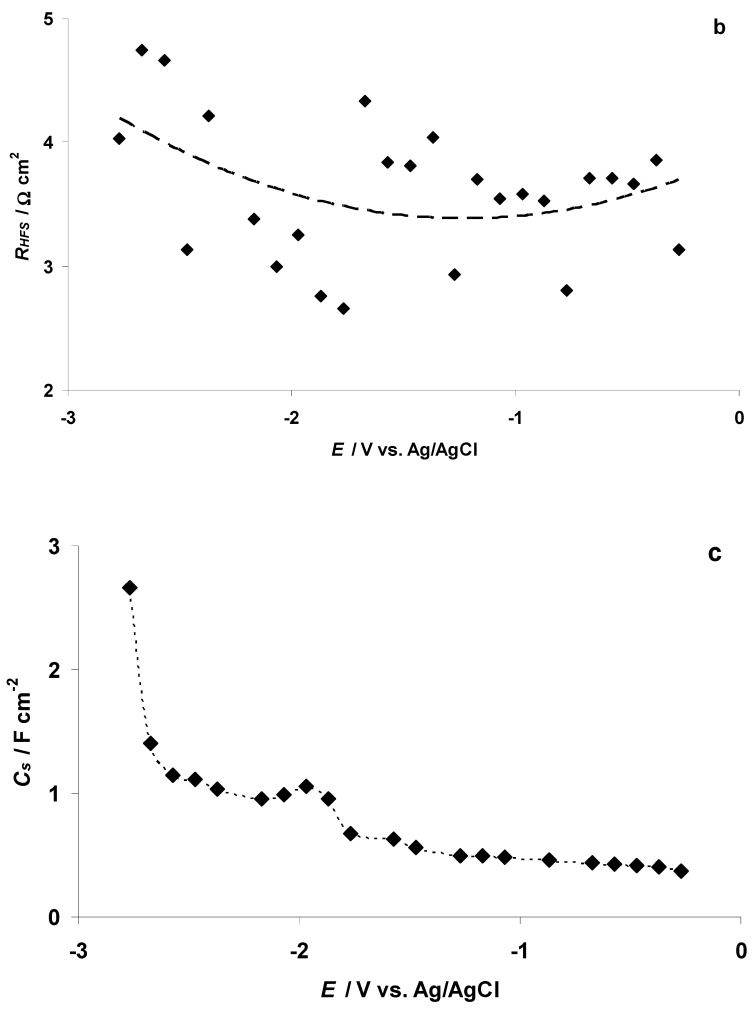

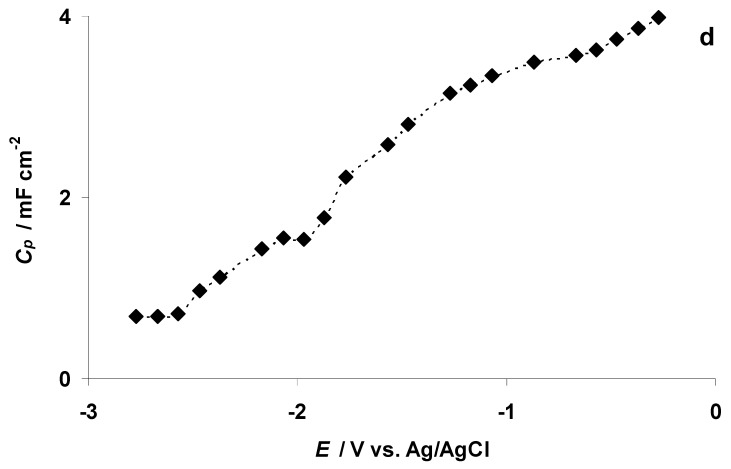
Illustrative data obtained from electrochemical impedance spectroscopy measurements: (**a**) series resistance (*R_s_*); (**b**) high frequency semicircle resistance (*R_HFS_*); (**c**) series capacitance (*C_s_*) (calculated at EIS modulation frequency (*ν*) *ν* = 0.1 Hz); (**d**) parallel capacitance (*C_p_*) (calculated at *ν* = 0.1 Hz) for different consecutively measured electrochemical impedance spectra at various negative potentials for C(Mo_2_C) in the 5 wt % EMImBr solution in EMImBF_4_.

Analysis of the Nyquist plots shows that at *E* ≤ −1.87 V the series resistance (*R_s_*, estimated from the Nyquist plot data) starts to increase (Figure 9a). The increase in *R_s_* is a clear indication of the formation of the EMIm–EMIm dimer dielectric film at the C(Mo_2_C) electrode surface. It is interesting to note that the resistance of the mass transport process in the electrode micro-mesoporous matrix (i.e., the width of the high-frequency semicircle, *R_HFS_*, calculated from the Nyquist plot data) remained almost stable in the potential range from −2.77 to −0.27 V (Figure 9b). The series capacitance *C_s_* (*C_s_* = −(*Z*″ 2*πν*)^−1^, where *ν* is the modulation frequency in Hz), calculated at 0.1 Hz, is stable until *E* = −1.77 V (Figure 9c). A small increase in the *C_s_* values then takes place parallel to the start of the electrochemical reduction of the EMIm^+^ cations. *C_s_* is maximal at *E* = −1.97 V, after which a small decrease in the *C_s_* values takes place (Figure 9c). At *E* ≤ −2.67 V, a very steep increase in *C_s_* values was observed, indicating that quick faradic processes take place at the C(Mo_2_C) surface (Figure 9c).

The parallel capacitance *C_p_* values (*C_p_* = −*Z*″ (|*Z*|^2^ 2*πν*)^−1^, where |*Z*| is impedance modulus), calculated at 0.1 Hz, decrease in a monotonous way during the increase in the negative polarization in the 5 wt % EMImBr solution in the EMImBF_4_–C(Mo_2_C) interface (Figure 9d). Some stabilization in the *C_p_* values (formation of the dielectric layer with low dielectric constant value) can be observed within the range −2.07 V < *E* < −1.97 V and at *E* ≤ −2.57 V (parallel to the increase in the *C_s_* values).

### 3.3. Characteristic Changes in the C 1s, N 1s, B 1s, F 1s, and Br 3d In Situ X-ray Spectra Collected at the Positively Polarized C(Mo_2_C) Electrode

**Figure 10:**
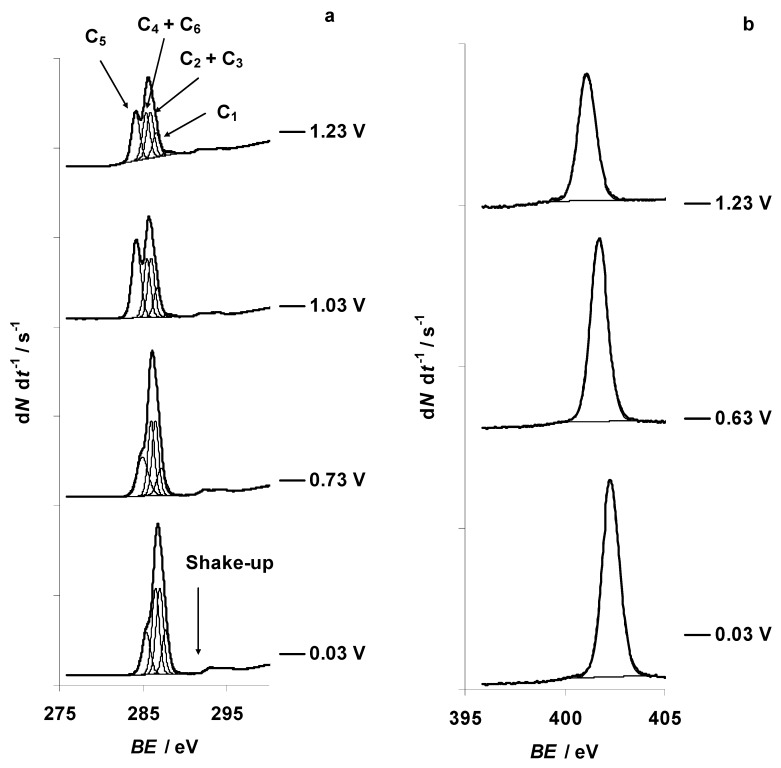

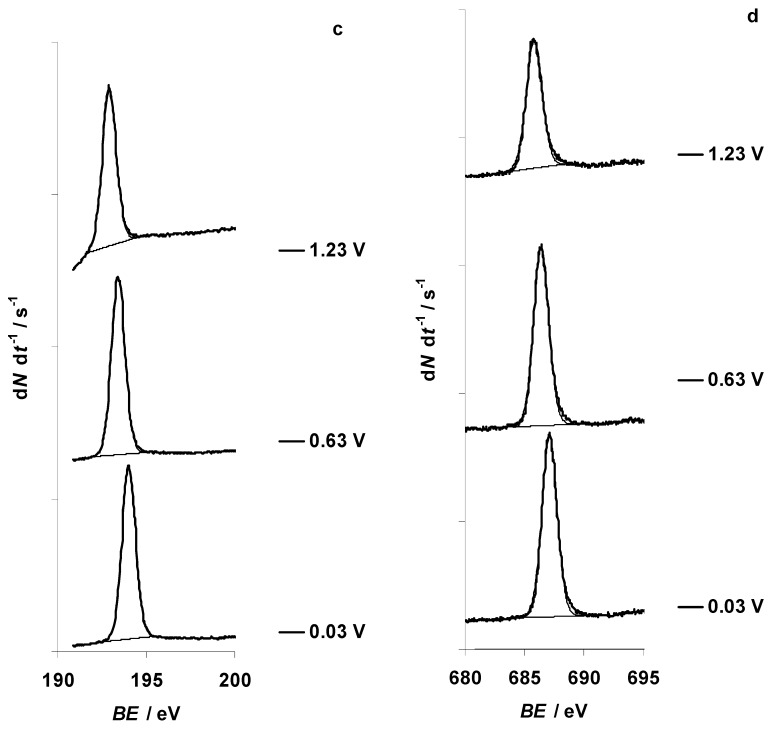

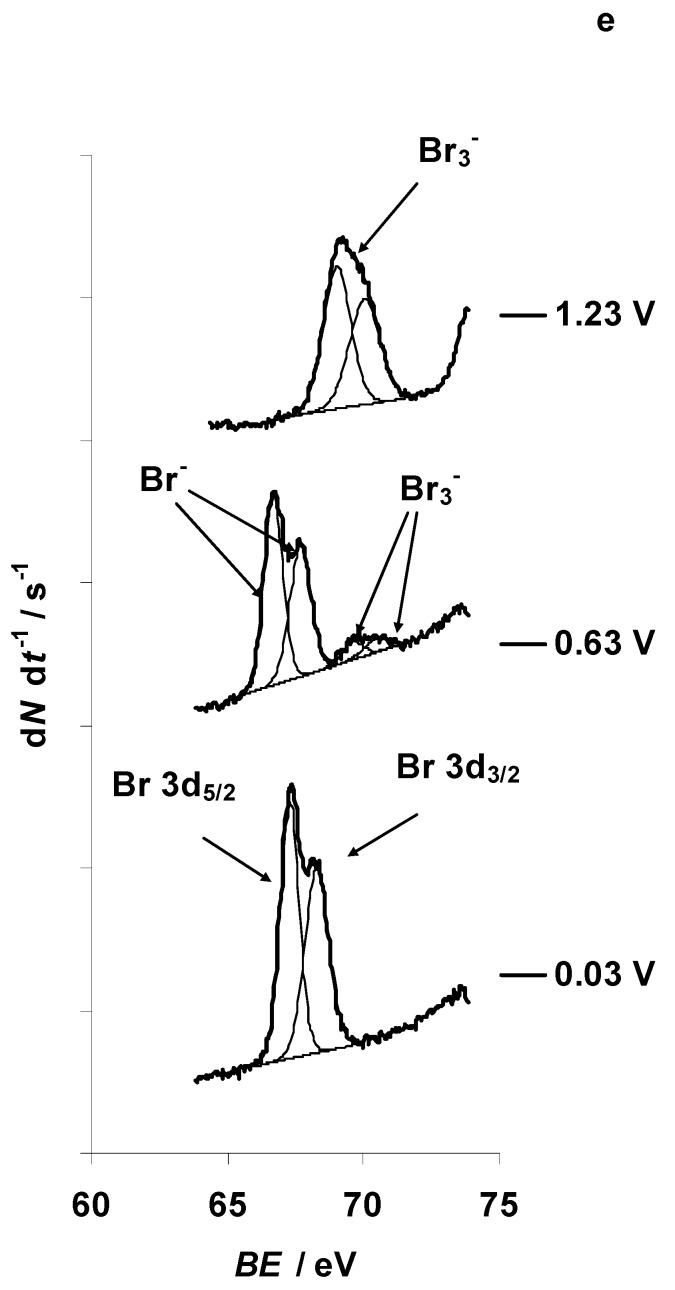
C 1s, N 1s, B 1s, F 1s, and Br 3d X-ray photoelectron (PE) spectra for the 5 wt % EMImBr solution in the EMImBF_4_ mixture measured at the fixed positive potentials of the C(Mo_2_C) electrode, noted in the figure. The binding energy (*BE*) scales for the XPS experiments have been referenced to the *BE* of the C 1s photoemission line related to aliphatic carbon (*BE* = 285.3 eV) measured for the non-polarized and grounded electrode: (**a**) C 1s PE spectra, fitted applying the four C 1s photoelectron peak model (regular lines); excitation energy was 400 eV, and the PE signal intensity (d*N* d*t*^−1^) scale between tick marks was 500 counts s^−1^; (**b**) N 1s PE spectra; excitation energy was 500 eV, and the PE signal intensity scale between tick marks was 200 counts s^−1^; (**c**) B 1s PE spectra; excitation energy was 250 eV, and the PE signal intensity scale between tick marks was 200 counts s^−1^; (**d**) F 1s PE spectra; excitation energy was 800 eV, and the PE signal intensity scale between tick marks was 10 counts s^−1^; (**e**) Br 3d PE spectra; excitation energy was 120 eV, and the PE signal intensity scale between tick marks was 50 counts s^−1^.

**Figure 11:**
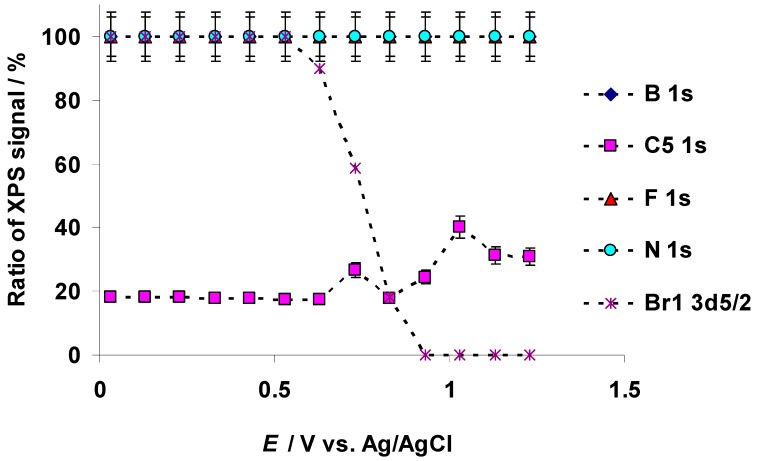
Dependence of EMIm^+^ “aliphatic” carbon C 1s (marked in the figure as C5), imidazolium nitrogen atoms N 1s, BF_4_^−^ anion boron B 1s, BF_4_^−^ anion fluorine F 1s, and Br^−^ anion bromine Br 3d_5/2_ (marked in the figure as Br1 3d5/2) photoelectron peaks ratios for the 5 wt % EMImBr solution in the EMImBF_4_ mixture at various positive potentials of the C(Mo_2_C) electrode.

The X-ray photoelectron spectra for C 1s, N 1s, B 1s, F 1s, and Br 3d PE-s at specific characteristic positive potentials were recorded within the potential range from −0.27 to 1.23 V and are presented in Figure 10a–e, respectively. Positions of the PE peaks, shown in Figure 10a–e, are presented in Table S3. The PE spectra indicate a change for the aliphatic carbon (C_5_) C 1s content at *E* = 0.73 V (Figure 10a), and the aliphatic carbon (C_5_) XPS signal ratio increased from its normal value X_C5_ ≈ 17% (Figure 11). The ratio of the C_5_ XPS signal increased significantly at *E* = 0.93 V, obtaining a peak value X_C5_ = 40% at *E* = 1.03 V (Figure 11). Thereafter, the ratio of C_5_ XPS signal stabilizes at X_C5_ = 31% at *E* ≥ 1.13 V. The in situ XPS measurements were stopped at *E* = 1.23 V due to the complete loss of the initial Br 3d_5/2_ signal, corresponding to the final electrooxidation and disappearance of Br^−^ ions (EMImBr) at the C(Mo_2_C) surface (discussed hereafter).

Br 3d_5/2_ and Br 3d_3/2_ PE peaks are notable at the positions *BE* = 67.3 eV and *BE* = 68.3 eV, respectively (at *E* = 0.03 V) (Figure 10e). The shape of the XPS spectrum started to change at *E* ≥ 0.63 V, when new Br 3d_5/2_ and Br 3d_3/2_ PE peaks formed at *BE* = 69.6(5) eV and *BE* = 70.5(5) eV, respectively (Figure 10e). Parallel to the expansion of new Br 3d PE peaks, the intensity of the initial Br 3d PE peaks (marked as Br1 in Figure 11), corresponding to the Br^−^ anion in the EMImBr, decreased (Figure 10e and Figure 11). At *E* ≥ 0.93 V, the initial Br 3d_5/2_ and Br 3d_3/2_ PE peaks disappeared and new ones appeared, originating (very probably) from the Br_3_^−^ complex anion signal. 

The recorded in situ XPS data indicate that the N 1s, B 1s, and F 1s PE signals were stable throughout the entire positive potentials region investigated (Figure 10b–d and Figure 11). Thus, these elements have not been involved in the electrochemical oxidation reactions.

### 3.4. The Electrochemical Measurements Data Collected at the Positively Polarized C(Mo_2_C) Electrode

**Figure 12:**
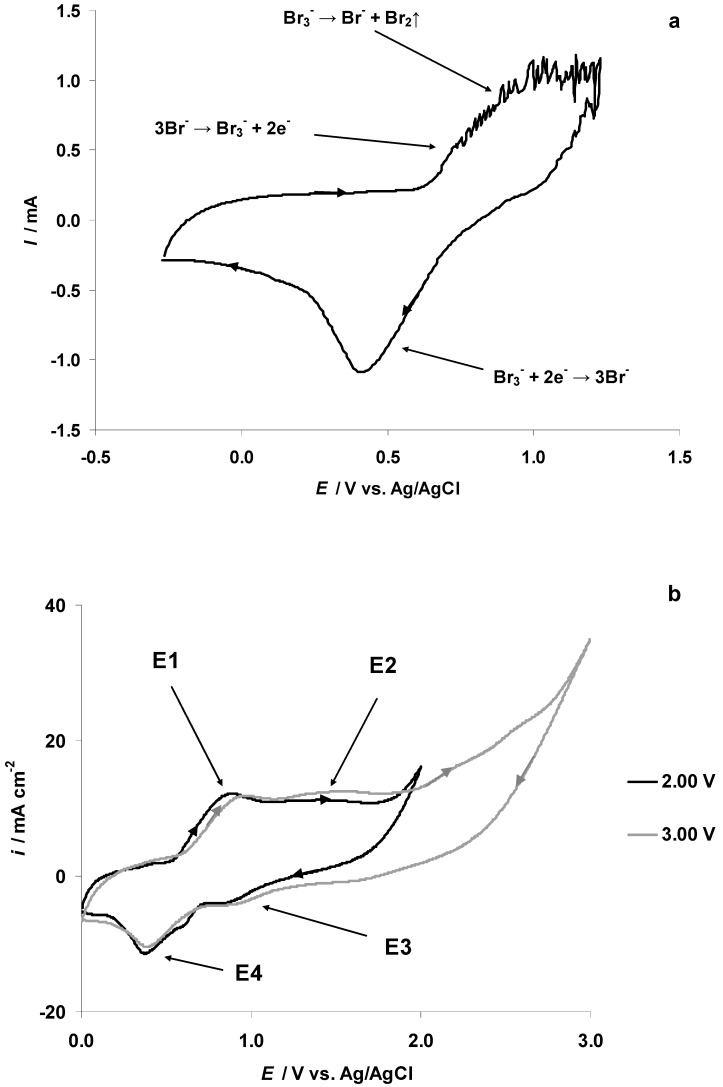
Cyclic voltammetry (CV) data for positively polarized C(Mo_2_C) electrodes soaked in 5 wt % EMImBr solution in the EMImBF_4_ solution: (**a**) located inside the XPS vacuum chamber (ca. *p* = 10^−7^ mbar) and (**b**) inside the very dry and oxygen free Ar-filled glovebox (ca. *p* = 1 bar) within the potential sweep ranges from 0.00 to 2.00 V and vice versa (black line) and from 0.00 to 3.00 V and vice versa (gray line). Data of second cycles have been presented measured at the potential scan rate of 1.0 mV s^−1^.

In order to understand the electrochemical behavior of the 5 wt % EMImBr solution in the EMImBF_4_–C(Mo_2_C) interface at positive potentials, CV measurements (with the potential sweep rate 1.0 mV s^−1^) were conducted at *p* ≈ 1 × 10^−7^ mbar after the end of the in situ XPS measurements within the potential range from −0.27 to 1.23 V and vice versa (Figure 12a, the second CV cycle is shown). The data show an almost exponential increase in the anodic current in the forward scan at *E* = 0.54 V due to the start of the Br^−^ anion electrooxidation to the Br_3_^−^ complex anion. However, it is not possible to observe the electrooxidation current peak, as the voltammogram became very noisy at *E* ≥ 0.74 V (Figure 12a) caused by the intensive electrooxidation of the Br^−^ anion and some increase in the intensity of the aliphatic carbon C_5_ 1s XPS PE peak signal (Figure 11). The formation of the Br_3_^−^ complex anion could not cause this kind of electrochemical noise, and we propose that the Br_3_^−^ complex anion was unstable in the high vacuum condition, dissociating to Br_2_ and Br^−^ anions. The formation of the Br_2_ gas at *E* ≥ 0.73 V could explain the increase in the XPS vacuum chamber pressure notable in Figure 5a. (However, the sudden reduction of the XPS chamber pressure at *E* > 1.13 V (Figure 5a), could be explained by the remarkable decrease in the Br^−^ anion concentration in the very thin electrolyte layer exposed to the exciting X-ray beam (Figure 11)) While Br_2_ has relatively high vapor pressure (at *T* ≈ 22 °C), the movement of Br_2_ gas bubbles from the inner part of the micro-mesoporous C(Mo_2_C) electrode to its surface and their collapse at the WE surface could explain the electrochemical noise in the CVs (Figure 12a). It should be noted that this kind of noise was not observed in the CVs recorded at *p* ≈ 1 bar, where the gas evolution from the electrode surface (i.e., “bubbling”) was less intense.

The CVs measured for the micro-mesoporous C(Mo_2_C) electrode in the 5 wt % EMImBr solution in EMImBF_4_ within the potential sweep range from 0.00 to 2.00 V (black line) and vice versa and from 0.00 to 3.00 V (gray line) and vice versa, as shown in Figure 12b. A first current maximum forms at *E* = 0.86 V, corresponding to the electrooxidation of the Br^−^ anion to the Br_3_^−^ complex (indicated as E1 in Figure 12b). At ca. *E* = 1.42 V, a wide voltammetric wave was observed (marked as E2 in Figure 12b). It is possible that sharp electrooxidation current peaks are not notable due to the large energetical inhomogenity of the micro-mesoporous C(Mo_2_C) electrode surface. Voltammetric waves of reduction processes, indicated in Figure 12b at *E* = 0.80 V as E3 and at *E* = 0.37 V as E4, were found. 

It should be noted that the separation between the 3Br^−^ → Br_3_^−^ + 2e^−^ and 2Br_3_^−^ → 3Br_2_ + 2e^−^ processes depends significantly on the electrolyte solution used [51]. The study of Allen et al. [51] showed that the stability of the Br_3_^−^ complex in 1-buthyl-3-methylimidazolium bis(trifluoromethylsulfonyl)imide (BMIm(NTf_2_)) was ca. 3000 times lower than that in the acetonitrile solution. The electrochemical oxidation of the Br^−^ anion to the Br_3_^−^ complex at the platinum electrode in BMIm(NTf_2_) was much slower (and irreversible) than at the platinum electrode soaked in an acetonitrile electrolyte [51]. On the other hand, Bennett et al. [52] demonstrated good separation, i.e., a ca. 0.5 V difference, between two consequent electrooxidation processes—3Br^−^ → Br_3_^−^ + 2e^−^ and 2) 2Br_3_^−^ → 3Br_2_ + 2e^−^—that take place at the glassy carbon electrode soaked in a 10 mM tetraethylammonium bromide solution in nitrobenzene.

Extending the CV sweep range up to 3.00 V (gray line, Figure 12b), the current density started to slowly increase at *E* > 1.95 V and new, low-intensity waves appeared at ca. *E* = 2.25 V and *E* = 2.60 V. However, due to the very low rate of the 2Br_3_^−^ → 3Br_2_ + 2e^−^ reaction at the glassy carbon electrode, the exact start of this electrooxidation reaction can not be established.

**Figure 13:**
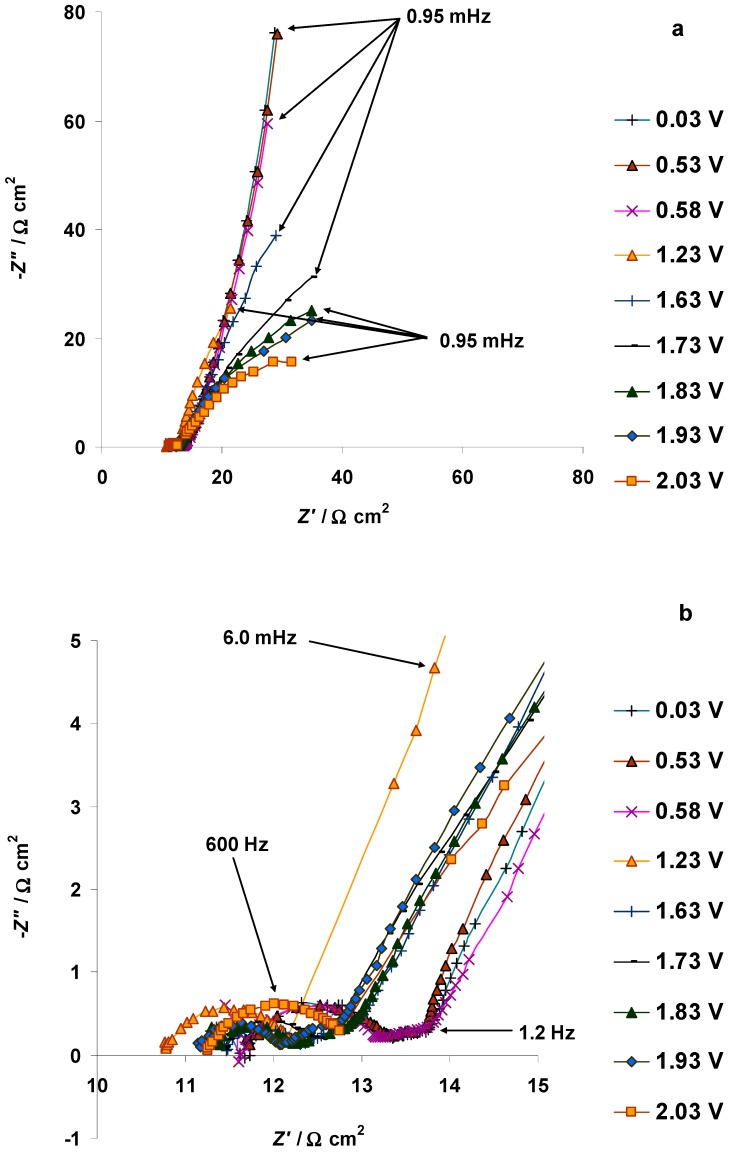

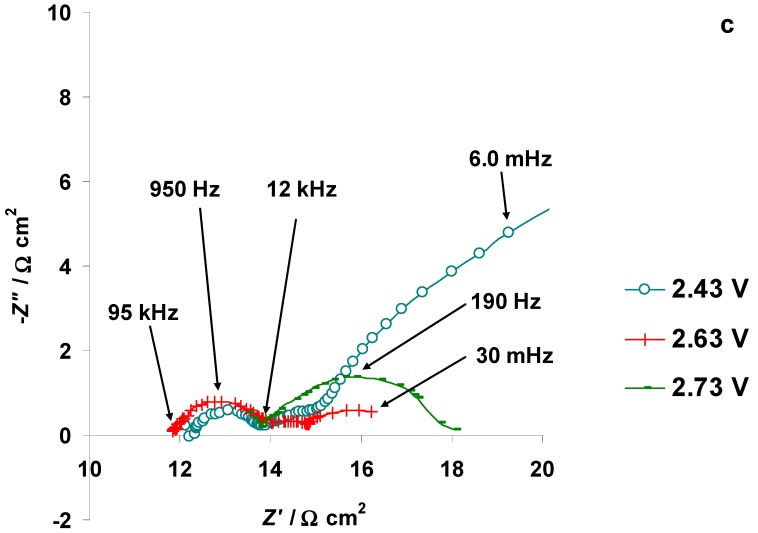
Electrochemical impedance spectroscopy Nyquist plots measured for the 5 wt % EMImBr solution in the EMImBF_4_–C(Mo_2_C) system at variable positive potentials: (**a**) from 0.03 to 2.03 V; (**b**) in the same potential region, but with the high frequency part extended; (**c**) at selected higher potentials, noted in the figure. *Z*′ and *Z*″ mark the real and imaginary parts of the electrochemical impedance, respectively.

Parallel with the CV measurements, EIS measurements in the potentiostatic regime (from *E* = 0.03 V up to *E* = 2.03 V) and within the frequency range from 300 kHz to 0.95 mHz were performed (Figure 13a). The –*Z*″ vs. *Z*′ plots overlap within the potential range from *E* = 0.03 to *E* = 0.53 V. The plot measured at *E* = 0.58 V has the same shape as the previous one, measured at *E* = 0.53 V. However, the *Z*″ value, measured at *E* = 0.58 V and ac frequency *ν* = 0.95 mHz, increased to −59.4 Ω cm^2^, compared to the *Z*″ value of −75.9 Ω cm^2^, obtained at *E* = 0.53 V and *ν* = 0.95 mHz. This could be read as an early indication of the start of the electrooxidation of the Br^−^ anion (Figure 11 and Figure 12a,b). Increasing the 5 wt % EMImBr solution in the EMImBF_4_–C(Mo_2_C) interface potential, the –*Z*″ vs. *Z*′ plot in the low frequency range preserves up to *E* = 1.73 V (Figure 13a). It is interesting to note that the high frequency semicircles are present throughout the potential range studied (0.03 V < *E* < 2.03 V), indicating that the micro-mesoporous structure of the C(Mo_2_C) electrode has not been blocked with the Br^−^ anion electrooxidation products (Figure 13b).

The Nyquist plot, measured at *E* = 2.63 V, contains high- and mid-frequency semicircles, and a low-frequency arc that indicate the intensification of the electrochemical oxidation processes in the 5 wt % EMImBr solution in the EMImBF_4_–C(Mo_2_C) interface (Figure 13c). The formation of an additional mid-frequency semicircle and a low-frequency arc corresponded to the very low-intensity wave at *E* = 2.60 V in CV in Figure 12b. The high-frequency semicircle disappeared and a low-frequency semicircle formed in the Nyquist plot measured at *E* = 2.73 V (Figure 13c). This indicates the complete blockage of the micro-mesoporous and slow charge transfer at the C(Mo_2_C) electrode surface. 

**Figure 14:**
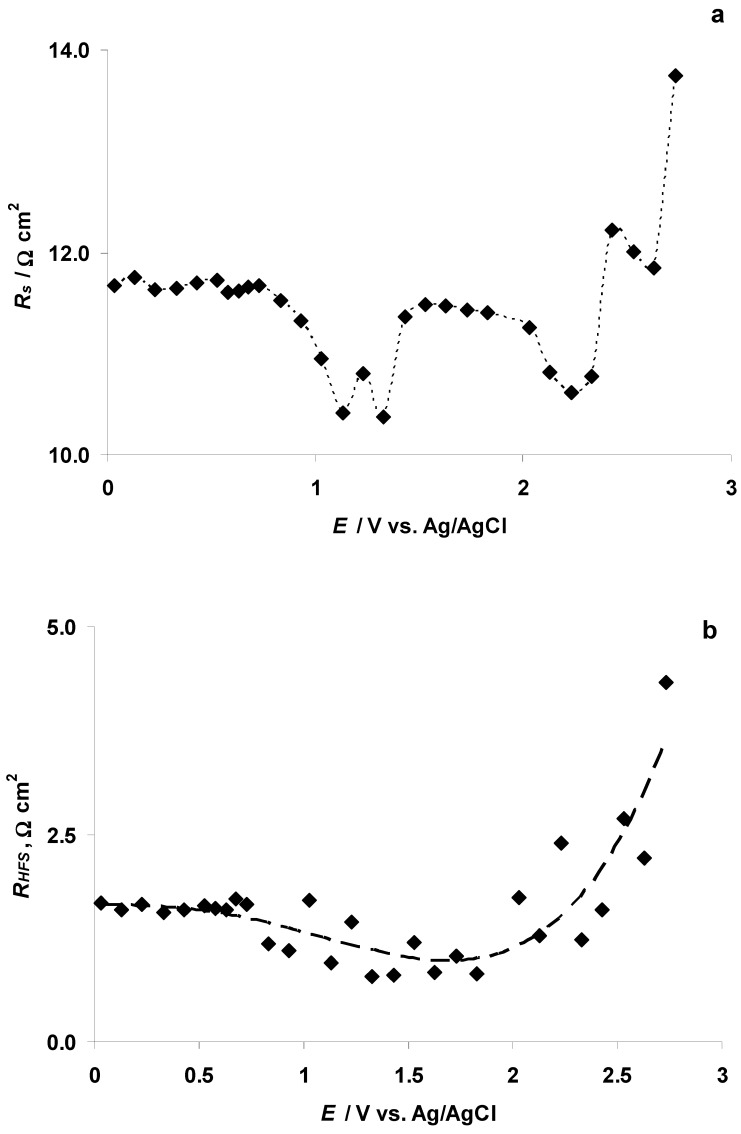

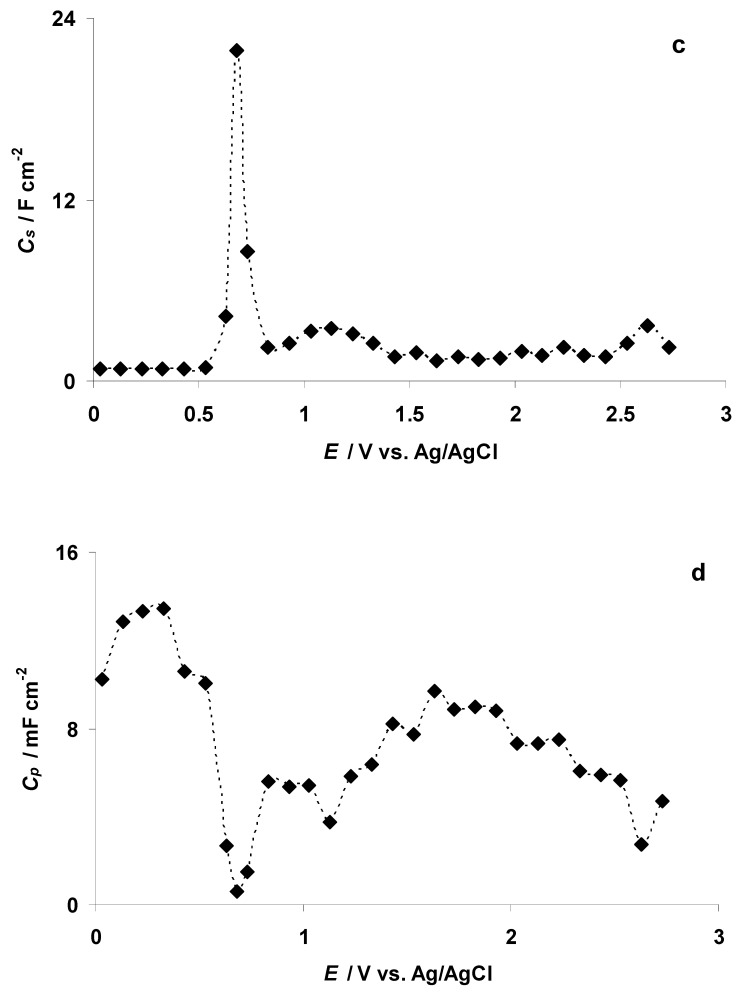

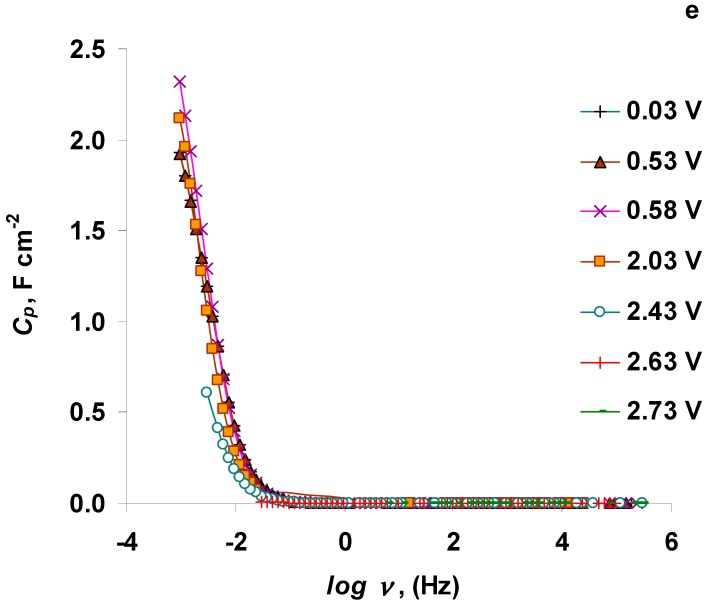

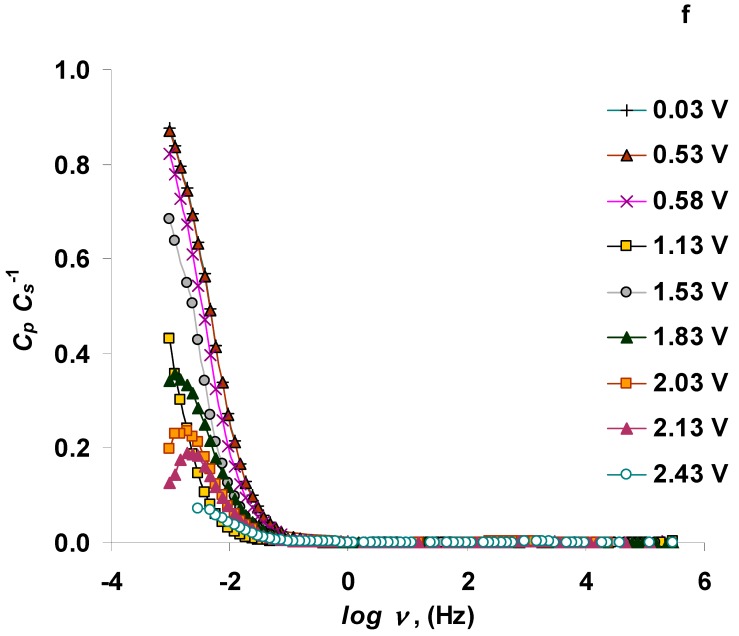
Illustrative data obtained from electrochemical impedance spectroscopy measurements: (**a**) —series resistance (*R_s_*); (**b**) high frequency semicircle resistance (*R_HFS_*); (**c**) series capacitance (*C_s_*) (calculated at EIS modulation frequency (*ν*) *ν* = 100 mHz); (**d**) parallel capacitance (*C_p_*) (calculated at *ν* = 100 mHz) for different consecutively measured electrochemical impedance spectra at various 5 wt % EMImBr solutions in the EMImBF_4_–C(Mo_2_C) system (i.e., C(Mo_2_C) electrode) positive potentials; (**e**) parallel capacitance (*C_p_*) vs. log *ν* (*ν* marks the modulation frequency in Hz) relationship for various 5 wt % EMImBr solutions in the EMImBF_4_–C(Mo_2_C) system positive potentials; (**f**) *C_p_ C_s_*^−1^ vs. log *ν* (*ν* marks the modulation frequency in Hz) relationship for various 5 wt % EMImBr solutions in the EMImBF_4_–C(Mo_2_C) system at positive potentials.

The *R_s_* values for the 5 wt % EMImBr solution in the EMImBF_4_–C(Mo_2_C) system estimated from the Nyquist plot data were stable (ca. *R_s_* = 12 Ω cm^2^), within the potential range 0.03 V < *E* < 0.73 V (Figure 14a). At *E* = 0.83 V, *R_s_* started to decrease, parallel to the very intensive electrooxidation of the Br^−^ anion, and a minimum value (ca. 10 Ω cm^2^) at *E* = 1.13 V (E1 in Figure 12b) was observed.

The *R_HFS_* (i.e., the mass transport resistance in the micro-mesoporous C(Mo_2_C) electrode pores) values are in agreement with the *R_s_* values at *E* < 0.83 V (Figure 14b). However, the R_HFS_ values became unstable at *E* > 0.73 V if the intensive electrooxidation of the Br^−^ anion to the Br_3_^−^ complex anion and Br_2_ (Figure 11 and Figure 12a,b) was observed. The increase in the *R_HFS_* at *E* > 1.8 V indicates a more restricted mass transport in the C(Mo_2_C) electrode pores.

*C_s_* values, calculated at *ν* = 0.1 Hz from the EIS measurements, show an intensive *C_s_* peak (*C_s_* ≈ 22 F cm^−2^) at *E* = 0.68 V (Figure 14c). The potential of the *C_s_* peak overlaps with the maximum rate of the Br^−^ anion electrooxidation to the Br_3_^−^ complex anion at micro-mesoporous C(Mo_2_C) (defined as E1 in Figure 12b and the C microelectrode (Figure 14c,d)). The *C_p_* values, calculated at *ν* = 0.1 Hz from the EIS measurements, are minimal at the same potentials, where *C_s_* has the maximum value, and intensive charge transfer processes, probably giving dielectric adsorbing products, take place (Figure 14d). *C_p_* vs. log *ν* data show that the *C_p_* values expand monotonously at log *ν* < −1.5 (Hz) (Figure 14e). The shape of the *C_p_* vs. log *ν* curves indicates the existence of a slow adsorption process inside the micro-mesoporous C(Mo_2_C) electrode. 

**Figure 15:**
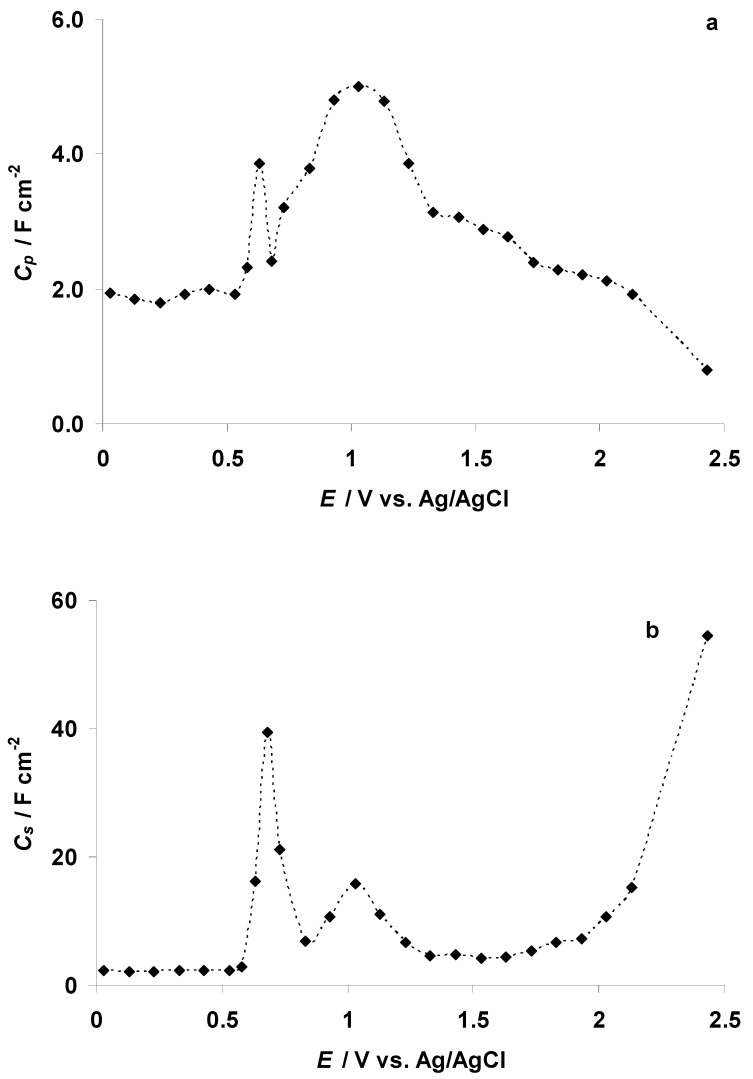
Data obtained from electrochemical impedance spectroscopy measurements: (**a**) parallel capacitance (*C_p_*) and (**b**) series capacitance (*C_s_*) values (calculated at *ν* = 0.95 mHz) for the 5 wt % EMImBr solution in the EMImBF_4_–C(Mo_2_C system at various positive potentials.

However, at higher C(Mo_2_C) potentials (*E* > 1.33 V) *C_p_* values (measured at *ν* = 0.95 mHz) decrease (Figure 15a), but *C_s_* values increase slightly (Figure 15b), indicating the intensification of the electrooxidation processes in the 5 wt % EMImBr solution in the EMImBF_4_–C(Mo_2_C) interface. At *E* ≥ 2.43 V, the Nyquist plots became unstable, so the calculation of *C_p_* was impossible.

The ratio *C_p_ C_s_*^−1^ = 0.9 (Figure 14f), calculated at *E* = 0.03 V (*ν* = 0.95 mHz), deviates from the value 1.0. The value 1.0 marks the ideal adsorption-limited process. Increasing the 5 wt % EMImBr solution in the EMImBF_4_–C(Mo_2_C) interface potential, the *C_p_ C_s_^−^*^1^ value decreased remarkably, indicating the existence of some very slow charge transfer reaction(s) at the electrode surface. At *E* = 1.83 V, a peak formed at *ν* = 1.2 mHz in the *C_p_ C_s_^−^*^1^ vs. log ν plot (*C_p_ C_s_*^−1^ = 0.4 at maximum peak. Continuing to increase the C(Mo_2_C) electrode potential toward more positive values, the value of *C_p_ C_s_*^−1^ and the maximum of the *C_p_ C_s_*^−1^ vs. log *ν* curve moved toward higher frequency values (*C_p_ C_s_*^−1^ = 0.2, at *E* = 2.03 V and *ν* = 1.9 mHz). 

The phase angle vs. *E* plot (Figure S1), obtained at *ν* = 0.1 Hz, had a similar shape as *C_p_* vs. *E* and *C_p_ C_s_*^−1^ vs. *E* plots (Figure 14e,f). It is notable that at *ν* = 0.1 Hz the phase angle values at all positive potentials were very low. The phase angle vs. *E* plot (Figure S2), obtained at *ν* = 0.95 mHz, had a shape similar to the *C_p_ C_s_*^−1^ vs. *E* relationship (Figure S3). It should be noted that the phase angle values, measured at *ν* = 0.95 mHz, have much more negative values than those obtained at *ν* = 0.1 Hz (Figure S1). Increasing the 5 wt % EMImBr solution in the EMImBF_4_–C(Mo_2_C) potential toward more positive values, the maximum phase angle value of −71.0° was recorded at *E* = 0.23 V (Figure S2). 

**Figure 16:**
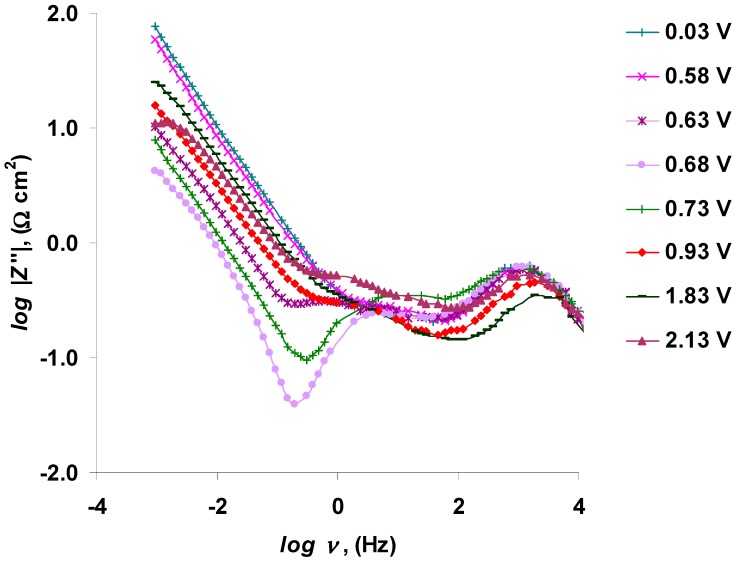
Electrochemical impedance spectroscopy (EIS) data: log of the imaginary part of impedance (*Z*″) vs. log of EIS modulation frequency (*ν*) dependences for the 5 wt % EMImBr solution in the EMImBF_4_–C(Mo_2_C) interface at various positive potentials, noted in the figure.

The log |*Z*″| vs. log *ν* data (Figure 16) indicate that the linear relationship exists only at very low frequencies (*ν* < 0.30 Hz). The slope and the length in the linear part of these plots depend somewhat on the potential applied. The slopes of the linear parts of the log |*Z*″| vs. log *ν* data are in the range from −0.8 to −0.7 (at 0.03 V < *E* < 2.33 V), indicating that mixed kinetic oxidation/adsorption processes prevail at the micro-mesoporous C(Mo_2_C) electrode within this potential region.

## 4. Conclusions

The in situ X-ray photoelectron spectroscopy (XPS) data for aliphatic carbon (C_5_) C_5_ 1s, N 1s, B 1s, F 1s, and Br 3d_5/2_ were measured for a 5 wt % 1-ethyl-3-methylimidazolium bromide solution in the 1-ethyl-3-methylimidazolium tetrafluoroborate–molybdenum carbide-derived carbon electrode interface at a residual water (210 ppm) level. The calculated data indicated that the 1s electrons binding energy vs. potential (d*BE* d*E*^−1^) plots for C, N, B, and F elements were all linear with the slope d*BE* d*E*^−1^ = −1 eV V^−1^ within the potential range from −1.17 to 1.23 V (i.e., in the region of ideal polarization). At more negative potentials (−2.07 V < *E* < −1.17 V), the d*BE* d*E*^−1^ value was nearly −0.5 eV V^−1^ for C_5_ 1s, N1 1s, B1 1s, F 1s, and Br 3d_5/2_ PEs. It was established that the reduction of the d*BE* d*E*^−1^ slope’s absolute value, twice at *E* ≤ −1.17 V, was connected with the start of the formation of gas bubbles at the C(Mo_2_C) electrode. The formation of a new B 1s PE peak, corresponding to the B–O bond, was caused by the electroreduction of the residual water adsorbed at the micro-mesoporous C(Mo_2_C) electrode.

The cyclic voltammetry (CV) measurements, performed in high vacuum conditions (ca. *p* = 10^−7^ mbar), indicated that the electrooxidation of the Br^−^ anion started at *E* = 0.54 V in the 5 wt % EMImBr solution in the EMImBF_4_–C(Mo_2_C)) interface. At *E* ≥ 0.74 V, the measured cyclic voltammogram became very noisy, indicating the instability of the Br_3_^−^ complex under vacuum (and the evaporation of Br_2_). The Br 3d_5/2_ XPS data indicated that the intensity of the Br^−^ anion electrooxidation at *E* ≥ 0.63 V (as the arbitrary intensity of the corresponding photoelectron (PE) peak) started to reduce and even disappeared at *E* ≥ 0.93 V. Parallel to the start of the decrease in the initial Br 3d_5/2_ and Br 3d_3/2_ PE peaks at *E* = 0.63 V, new Br 3d_5/2_ and 3d_3/2_ PE peaks (at ca. Δ*BE* = 3 eV higher *BE*s) formed, corresponding to the formation of the Br_3_^−^ complex anion.

The CV method was not sensitive enough to separate the 3Br^−^ → Br_3_^−^ + 2e^−^ and 2Br_3_^−^ → 3Br_2_ + 2e^−^ processes taking place in the 5 wt % EMImBr solution in the EMImBF_4_–micro-mesoporous C(Mo_2_C) interface. However, separation and quantitative analysis of these electrochemical reactions is possible based on Br 3d_5/2_ in situ XPS and electrochemical impedance data. On the other hand, CV measurements provided useful information for 5 wt % EMImBr solutions in an EMImBF_4_–carbon fiber microelectrode system due to the larger ratios of signal to noise and of faradic current to charging current.

## Supplementary Materials

The following are available online at https://www.mdpi.com/2079-4991/9/2/304/s1. Table S1: C 1s, N 1s, B 1s, F 1s, and Br 3d PE binding energies (eV) measured by in situ XPS for the 5 wt % EMImBr solution in the EMImBF_4_–C(Mo_2_C) system polarized at various negative potentials (corresponding spectra are shown in Figure 2a–e). Table S2: d*BE* vs. d*E* (eV V^−1^) slopes for aliphatic carbon (C_5_) C 1s, initial nitrogen (N1) N 1s, initial boron (B1) B 1s, F 1s, and initial bromine (Br) Br 3d_5/2_ photoelectron binding energies measured for the 5 wt % EMImBr solution in the EMImBF_4_–C(Mo_2_C) system for various potential ranges. Table S3: C 1s, N 1s, B 1s, F 1s, and Br 3d PE binding energies (eV) measured by in situ XPS for the 5 wt % EMImBr solution in the EMImBF_4_–C(Mo_2_C) system polarized at various positive potentials (corresponding spectra are shown in Figure 10a–e). Figure S1: Electrochemical impedance spectroscopy phase angle data for the 5 wt % EMImBr solution in EMImBF_4_, measured at *ν* = 0.1 Hz and at various C(Mo_2_C) electrode positive potentials. Figure S2: Electrochemical impedance spectroscopy phase angle data for the 5 wt % EMImBr solution in EMImBF_4_, measured at *ν* = 0.95 mHz and at various C(Mo_2_C) electrode positive potentials. Figure S3: The parallel capacitance (*C_p_*) and series capacitance (*C_s_*) ratio (*C_p_ C_s_*^−1^) data at various C(Mo_2_C) electrode positive potentials and *ν* = 0.95 mHz, obtained for the 5 wt % EMImBr solution in the EMImBF_4_–C(Mo_2_C) interface.
