# Supplementary material for: In Situ X-ray Photoelectron Spectroscopic and Electrochemical Studies of the Bromide Anions Dissolved in 1-Ethyl-3-Methyl Imidazolium Tetrafluoroborate
Source: Nanomaterials (Basel). 2019 Feb 22;9(2):304. doi: 10.3390/nano9020304 (PMC6409973; doi:10.3390/nano9020304)
Supplement: Supplementary file 1 [file nanomaterials-09-00304-s001.pdf]

nanomaterials

Supplementary materials

In situ X-ray photoelectron spectroscopic and electrochemical studies of the bromide anions dissolved 1-ethyl-3-methylimidazolium tetrafluoroborate

Jaanus Kruusma [1], Arvo Tõnisoo [2], Rainer Pärna [2], Ergo Nõmmiste [2] and Enn Lust [1,*]

[1] Institute of Chemistry, University of Tartu, Ravila 14A, 50411 Tartu, Estonia; jaanus.kruusma@ut.ee (J.K.)
[2] Institute of Physics, University of Tartu, W. Ostwaldi 1, 50411 Tartu, Estonia; arvo.tonisoo@ut.ee (A.T.); rainer.parna@ut.ee (R.P.); ergo.nommiste@ut.ee (E.N.)
* Correspondence: enn.lust@ut.ee (E.L); Tel.: (+372-737-5165)

Table S1. C 1s, N 1s, B 1s, F 1s and Br 3d PE binding energies (eV) measured by *in situ* XPS for 5 wt% EMImBr + $EMImBF_4$ | $C(Mo_2C)$ system polarized at various negative potentials (corresponding spectra are shown in Figures. 2a – e).

C 1s	E = -0.27 V	E = -1.27 V	E = -2.07 V
C1	287.6(0)	288.4(0)	288.7(0)
C2 + C3	286.9(0)	287.7(0)	288.0(0)
C4 + C6	286.4(0)	287.2(0)	287.5(0)
C5	285.3(5)	286.1(5)	286.6(5)
N 1s	E = -0.27 V	E = -1.27 V	E = -2.07 V
N1	402.2(0)	402.9(5)	403.5(5)
N2	—	400.4(5)	400.9(0)
B 1s	E = -0.27 V	E = -1.17 V	E = -2.07 V
B1	194.0(0)	194.8(0)	195.2(5)
B2	—	193.3(5)	193.9(5)
F 1s	E = -0.27 V	E = -1.17 V	E = -2.07 V
F	686.8(0)	687.9(0)	688.2(5)
Br 3d	E = -0.27 V	E = -1.17 V	E = -2.07 V
Br $3d_{5/2}$	67.3(0)	68.0(5)	68.5(5)
Br $3d_{3/2}$	68.3(0)	69.1(0)	69.6(0)

Table S2. d*BE* vs. d*E* (eV V^{-1}) slopes for aliphatic carbon (C_5) C 1s, initial nitrogen (N1) N 1s, initial boron (B1) B 1s, F 1s and initial bromine (Br) Br $3d_{5/2}$ photoelectron binding energies measured for 5 wt% EMImBr + $EMImBF_4$ | $C(Mo_2C)$ system for various potential ranges.

	-2.03 V < E < -1.17 V	-1.17 V < E ≤ 1.23 V
(C_5) C 1s	-0.50 (R^2 = 0.970)	-0.98 (R^2 = 0.995)
(N1) N 1s	-0.52 (R^2 = 0.924)	-0.91 (R^2 = 0.997)
(B1) B 1s	-0.50 (R^2 = 0.952)	-0.92 (R^2 = 0.999)
F 1s	-0.42 (R^2 = 0.782)	-0.87 (R^2 = 0.979)
(Br1) Br $3d_{5/2}$	-0.52 (R^2 = 0.948)	-0.72 (R^2 = 0.970)*

* Calculated for the potential range -1.17 V < E < 0.73 V.

Table S3. C 1s, N 1s, B 1s, F 1s and Br 3d PE binding energies (eV) measured by *in situ* XPS for 5 wt% EMImBr + EMImBF$_4$ | C(Mo_2C) system polarized at various positive potentials (corresponding spectra are shown in Figures. 10a – e).

C 1s	*E* = 0.03 V	*E* = 0.73 V	*E* = 1.03 V	*E* = 1.23 V
C1	287.6(5)	287.1(5)	286.6(5)	286.5(5)
C2 + C3	286.9(5)	286.4(5)	285.9(5)	285.8(5)
C4 + C6	286.4(5)	285.9(5)	285.4(5)	285.3(5)
C5	285.3(5)	284.8(0)	284.1(5)	284.1(0)
N 1s	*E* = 0.03 V	*E* = 0.63 V	*E* = 1.23 V	
N	402.3(0)	401.7(0)	401.1(0)	
B 1s	*E* = 0.03 V	*E* = 0.63 V	*E* = 1.23 V	
B	194.0(0)	193.4(0)	192.9(0)	
F 1s	*E* = 0.03 V	*E* = 0.63 V	*E* = 1.23 V	
F	687.1(0)	686.5(0)	685.8(0)	
Br 3d	*E* = 0.03 V	*E* = 0.63 V	*E* = 1.23 V	
Br1 $3d_{5/2}$	67.3(0)	66.7(0)	—	
Br1 $3d_{3/2}$	68.3(0)	67.7(0)	—	
Br2 $3d_{5/2}$	—	69.6(0)	69.0(0)	
Br2 $3d_{3/2}$	—	70.4(0)	70.0(0)	

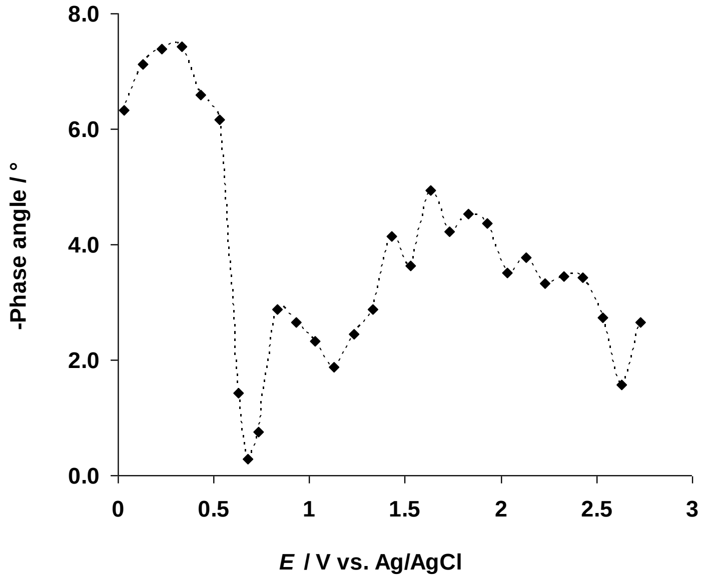

Figure S1. Electrochemical impedance spectroscopy phase angle data for 5 wt% EMImBr + EMImBF$_4$ solution, measured at ν = 0.1 Hz and at various C(Mo_2C) electrode positive potentials.

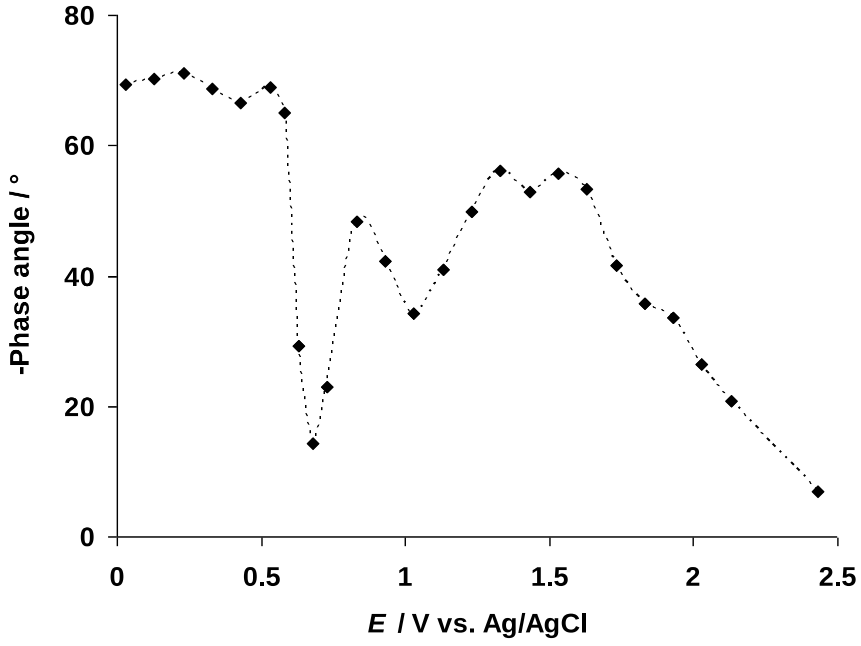

Figure S2. Electrochemical impedance spectroscopy phase angle data for 5 wt% EMImBr + $EMImBF_4$ solution, measured at $\nu = 0.95$ mHz and at various $C(Mo_2C)$ electrode positive potentials.

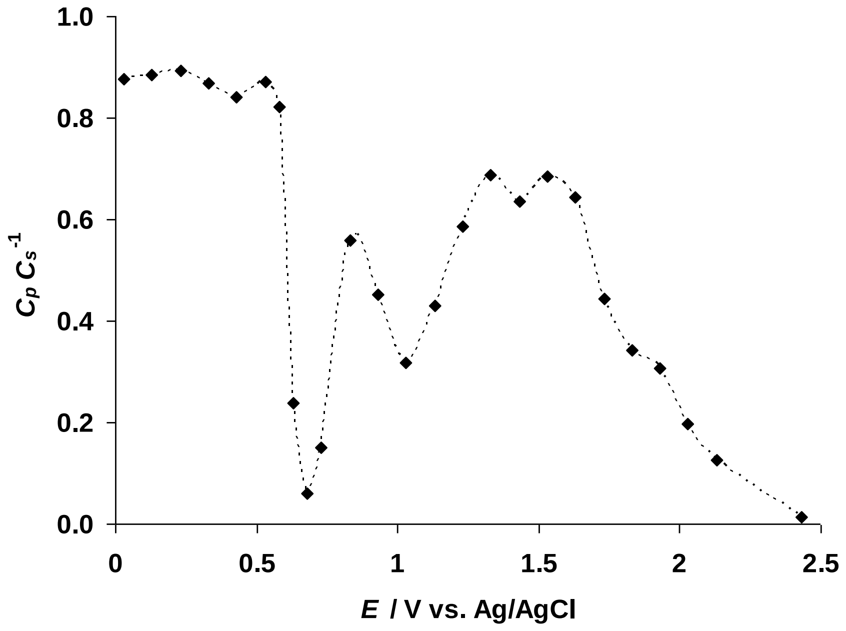

Figure S3. The parallel capacitance (C_p) and series capacitance (C_s) ratio (C_p C_s^{-1}) data at various $C(Mo_2C)$ electrode positive potentials and $\nu = 0.95$ mHz, obtained for 5 wt% EMImBr + $EMImBF_4$ | $C(Mo_2C)$ interface.

© 2019 by the authors. Submitted for possible open access publication under the terms and conditions of the Creative Commons Attribution (CC BY) license (http://creativecommons.org/licenses/by/4.0/).
